# A transcriptional cycling model recapitulates chromatin-dependent features of noisy inducible transcription

**DOI:** 10.1371/journal.pcbi.1010152

**Published:** 2022-09-09

**Authors:** M. Elise Bullock, Nataly Moreno-Martinez, Kathryn Miller-Jensen

**Affiliations:** 1 Department of Biomedical Engineering, Yale University, New Haven, Connecticut, United States of America; 2 Department of Molecular, Cellular, and Developmental Biology, Yale University, New Haven, Connecticut, United States of America; 3 Systems Biology Institute, Yale University, New Haven, Connecticut, United States of America; Heidelberg University, GERMANY

## Abstract

Activation of gene expression in response to environmental cues results in substantial phenotypic heterogeneity between cells that can impact a wide range of outcomes including differentiation, viral activation, and drug resistance. An important source of gene expression noise is transcriptional bursting, or the process by which transcripts are produced during infrequent bursts of promoter activity. Chromatin accessibility impacts transcriptional bursting by regulating the assembly of transcription factor and polymerase complexes on promoters, suggesting that the effect of an activating signal on transcriptional noise will depend on the initial chromatin state at the promoter. To explore this possibility, we simulated transcriptional activation using a transcriptional cycling model with three promoter states that represent chromatin remodeling, polymerase binding and pause release. We initiated this model over a large parameter range representing target genes with different chromatin environments, and found that, upon increasing the polymerase pause release rate to activate transcription, changes in gene expression noise varied significantly across initial promoter states. This model captured phenotypic differences in activation of latent HIV viruses integrated at different chromatin locations and mediated by the transcription factor NF-κB. Activating transcription in the model via increasing one or more of the transcript production rates, as occurs following NF-κB activation, reproduced experimentally measured transcript distributions for four different latent HIV viruses, as well as the bimodal pattern of HIV protein expression that leads to a subset of reactivated virus. Importantly, the parameter ‘activation path’ differentially affected gene expression noise, and ultimately viral activation, in line with experimental observations. This work demonstrates how upstream signaling pathways can be connected to biological processes that underlie transcriptional bursting, resulting in target gene-specific noise profiles following stimulation of a single upstream pathway.

## Author summary

Many genes are transcribed in infrequent bursts of mRNA production through a process called transcriptional bursting, which contributes to variability in responses between cells. Heterogeneity in cell responses can have important biological impacts, such as whether a cell supports viral replication or responds to a drug, and thus there is an effort to describe this process with mathematical models to predict biological outcomes. Previous models described bursting as a transition between an “OFF” state or an “ON” state, an elegant and simple mathematical representation of complex molecular mechanisms, but one which failed to capture how upstream activation signals affected bursting. To address this, we added an additional promoter state to better reflect biological mechanisms underlying bursting. By fitting this model to variable activation of quiescent HIV infections in T cells, we showed that our model more accurately described viral expression variability across cells in response to an upstream stimulus. Our work highlights how mathematical models can be further developed to understand complex biological mechanisms and suggests ways to connect transcriptional bursting to upstream activation pathways.

## Introduction

Heterogeneity in gene expression between cells impacts a wide range of phenotypic outcomes, including differentiation [1,2], viral expression [3–7], and drug resistance [8]. In eukaryotic cells, a major source of gene expression heterogeneity is transcriptional bursting [9–11], in which a gene promoter infrequently produces bursts of transcripts. Two metrics are often used to describe a gene’s burstiness: burst size, quantifying how many transcripts are produced in one burst, and burst frequency, describing how many bursts occur over time. Influencing transcriptional bursting, either through altering burst size or burst frequency, can aid in clinically relevant settings where transcriptional noise plays a role in disease progression [12].

Several possible points of control have been proposed for transcriptional bursting, including transcription factor (TF) regulation [13,14], polymerase recycling [15], chromatin environment [11,16], nucleosome positioning [17,18], and polymerase pause regulation [4,19]. Despite this complexity, transcriptional bursting is most often modeled as a simple random telegraph process, in which a promoter infrequently transitions from an “OFF” state to an “ON” state [20]. Regardless of this simplicity, the two-state model accurately reflects transcriptional bursting in many biological contexts [11,21]. To address situations in which the two-state model does not capture all aspects of transcriptional noise, additional layers of complexity have been added, including multiple “OFF” states [20,22,23], a continuum of states [24], and a refractory state [11,25,26].

One biological context in which the two-state promoter model lacks descriptive detail is in recapitulating the role of TF activation in inducible gene expression. While several studies have assessed how TFs modulate inducible gene expression noise [27,28], they are limited by an inability to directly connect molecular steps in transcription to changes in promoter state. TFs often recruit molecular complexes that alter aspects of both transcriptional burst size and burst frequency. For example, tumor necrosis factor (TNF) initiates a signaling cascade that activates the canonical TF nuclear factor-κB (NF-κB) [29]. NF-κB mediates recruitment of histone acetyltransferases (HATs), including CBP/p300 [30], to target promoters, destabilizing DNA-histone interactions within nucleosomes and increasing promoter accessibility. NF-κB also mediates recruitment of Mediator, RNA polymerase II (RNAPII) and other members of the preinitiation complex to accessible promoters [31]. Finally, NF-κB interacts with bromodomain-containing protein 4 (Brd4) to recruit and activate the positive elongation factor b (P-TEFb), releasing paused RNAPII and allowing efficient transcriptional elongation to proceed [32–34]. These processes are important for inducible transcription in many contexts including inflammatory gene expression [35] and latent HIV activation [4,36].

A recent model of transcriptional bursting that includes an additional promoter state to decouple RNAPII pausing from RNAPII recruitment more accurately describes steady-state transcriptional bursting [7,19]. Here, we explored if this three-state promoter model of transcription, which explicitly models the transcriptional steps of chromatin remodeling, polymerase recruitment, and polymerase pause release, could also capture features of noisy inducible transcription. Through deterministic and stochastic simulations, we explored parameter ranges for a three-state promoter model that describe chromatin environments of quiescent-but-inducible promoters in a range of biological contexts. We found that upon transcriptional activation implemented by increasing the polymerase pause release rate, changes in gene expression noise varied significantly across initial promoter states. We then fit our model simulations to time-resolved, single-cell experimental data of how NF-κB activation induces transcription of latent HIV viruses integrated into different chromatin environments. We found that the model accurately captured how exogenous stimulation of NF-κB differentially affected transcriptional noise and viral reactivation initiated in a range of basal states. Furthermore, the model allowed for exploration of the influence of multiple NF-κB-mediated steps in transcriptional activation. We anticipate this model will offer a means to connect transcriptional bursting to upstream signaling pathways, with applications that extend to other NF-κB-mediated biological systems, and potentially other signaling pathways.

## Results

### Steady-state analysis of a transcriptional cycling model reveals that distinct rate ratios control promoter accessibility versus transcriptional cycling

**Fig 1:**
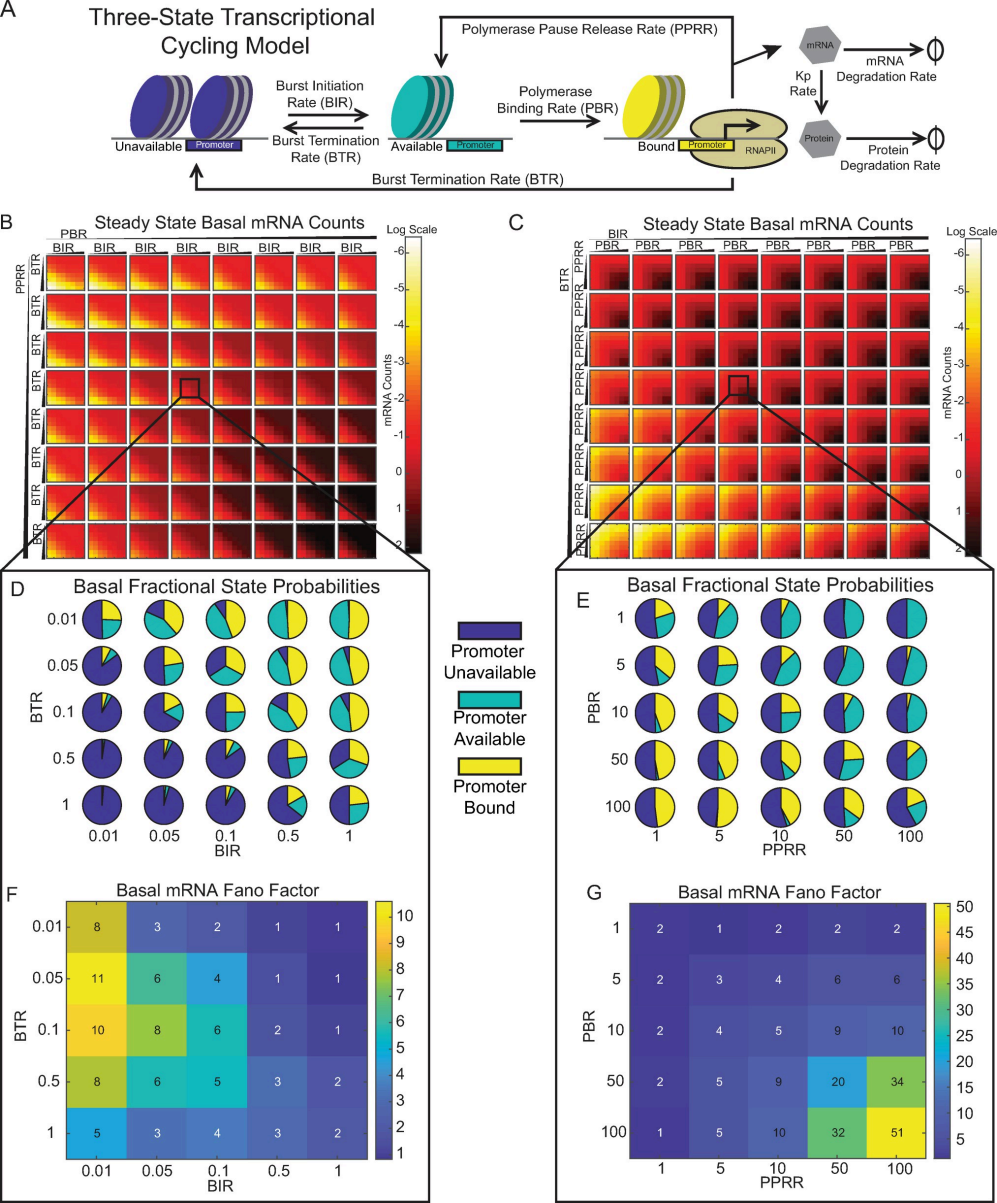
The BIR:BTR ratio controls promoter availability and the PPRR:PBR ratio controls transcriptional cycling. (A) Schematic of three-state transcriptional cycling model, including five species: an unavailable promoter (UP, in blue), an available (but unbound) promoter (AP, in teal), a bound promoter (BP, in yellow), RNA, and protein. A cycle of transcription occurs when the promoter transitions from BP to AP and back to BP. (B-C) Deterministic solution of steady-state mRNA counts when varying BIR and BTR for fixed values of PBR and PPRR (B) or when varying PBR and PPRR for fixed values of BIR and BTR (C). Parameter ranges are varied low to high via arrow directionality and correspond to the following sets: PBR = [0.1 0.5 1 5 10 50 100 500] hr^-1^, PPRR = [0.1 0.5 1 5 10 50 100] hr^-1^, BIR = [0.005 0.01 0.05 0.1 0.5 1 5 10 50] hr^-1^, and BTR = [0.005 0.01 0.05 0.1 0.5 1 5 10 50] hr^-1^. Heatmap indicates log of average mRNA levels. (D-E) Pie charts representing the fractional probability of UP (blue), AP (teal), and BP (yellow) when varying the BIR:BTR ratio for fixed PBR and PPRR (D) or when varying PBR:PPRR ratio for fixed BIR and BTR (E). (F-G) Fano Factor calculated for the same range of simulations presented in (D) and (E), respectively. Fractional promoter state probabilities and Fano factors were calculated from 1,000 single-cell stochastic simulations under basal conditions out to 10 days for each parameter combination. Square inset in (B) corresponds to the following parameter set: PBR = 10 hr^-1^, PPRR = 10 hr^-1^, BIR = [0.01 0.05 0.1 0.5 1] hr^-1^, and BTR = [0.01 0.05 0.1 0.5 1] hr^-1^. Square inset in (C) corresponds to the following parameter set: BTR = 0.1 hr^-1^, BIR = 0.1 hr^-1^, PPRR = [1 5 10 50 100] hr^-1^, and PBR = [1 5 10 50 100] hr^-1^.

To explicitly consider the effect of promoter accessibility and RNAPII pausing on transcriptional noise, we explored a previously published three-state promoter model [19]. In this model, transcription of a target gene is regulated by its promoter which transitions between three states, only one of which is productive for producing mRNA (Fig 1A). The rate at which the unavailable promoter (UP) state transitions to the available promoter (AP) state is the burst initiation rate (BIR) and the returning promoter transition rate is the burst termination rate (BTR). These two rates could be assumed to describe the remodeling of the chromatin environment surrounding the promoter that increases promoter availability [17,37], including nucleosome repositioning in the case of HIV [38]. The rate at which RNAPII and the associated transcriptional machinery binds to the AP state and transitions to the bound promoter (BP) state is the polymerase binding rate (PBR). In the BP state, which is assumed to describe an initiated but paused promoter [39], the rate at which the promoter releases an elongated transcript and returns to the AP state is the polymerase pause release rate (PPRR). Elongated transcripts are translated into protein at a rate Kp, and degradation of mRNA and proteins is modeled as a first-order process. We assume that only one polymerase can bind at a time, and that the polymerase remains paused until released [39,40]. If the promoter continuously cycles between the AP and BP states, then a burst of transcription occurs, and therefore we refer to this model as a transcriptional cycling model. Alternatively, the BP state returns to the UP state with the same BTR as the AP transitions to UP state, denoting a burst termination event. As in previous applications of this model, we assumed that the rate of transition to the UP state from either the AP or BP state is the same, because the biological processes regulating these transitions–namely, TF removal and chromatin remodeling–are similar to each other and distinct from the biological processes governing the other transition rates [7,19].

This transcriptional cycling model was shown to accurately reflect the biological regulatory mechanisms of burst initiation and pause release for steady-state gene expression [19]. At higher PPRR or PBR values, the exact solution of this model reduces to the two-state model, as the transition from unavailable promoter to available promoter would produce transcripts constitutively [7]. However, it is unclear how variability in inducible transcription would be affected when initiated under different steady-state conditions, which could reflect different chromatin environments.

To explore this, we used HIV as a model system. As a retrovirus, HIV integrates into the infected host cell’s genome [41]. Although most viral integrations lead to productive infections, a rare subset of integrated viruses transition to a latent state, in which little or no virus is actively produced [42]. There is no cure for HIV infection due to this latent reservoir, but quiescent viruses can be reactivated upon stimulation with certain extracellular cues, offering a clinically promising strategy to purge the latent viral reservoir via exploiting the molecular mechanisms that cause activation. The HIV long terminal repeat (LTR) promoter is regulated by NF-κB [43], and TNF stimulation leads to the accumulation of the NF-κB RelA:p50 heterodimer in the nucleus, leading to transcriptional activation [44]. Notably, HIV exhibits bursty transcription that is dependent on the chromatin environment at the location of viral integration [5,45], with more open chromatin environments leading to higher levels of activation [46,47].

We first sought to understand the parameter space of the three-state transcriptional cycling model (Fig 1A) by examining average steady-state solutions for HIV mRNA and promoter-state distributions calculated from the five ordinary differential equations (ODEs) in the model (see Materials and Methods). Degradation and translation rates were held constant at previously experimentally determined values for HIV [18,48,49], while the remaining four parameters were varied over a wide biological range (see Materials and Methods for explanation of this range). Examination of the 4-D parameter space revealed regions of varying fractional promoter state probabilities (S1A–S1C Fig). Higher BTR values increased the probability of promoters residing in the UP state (S1A Fig), whereas increases in BIR resulted in a higher probability of having promoters in the AP and BP states (S1B and S1C Fig). Increases in PPRR were associated with a higher probability of being in the AP state and increases in PBR increased the probability of being in the BP state given a certain UP probability (BIR to BTR). The clear patterns along the diagonal within each BIR-BTR square strongly suggested that the ratio of these parameters regulates the distribution of promoter states.

To support these qualitative observations, we derived quantitative relationships between parameter ratios and the ratios of promoter states by examining the five ODEs at steady-state. Overall, we found that

$$\frac{BIR}{BTR}=\frac{\left[AP]+[BP\right]}{\left[UP\right]}$$ Eq 1

indicating that the relative magnitude of BIR to BTR controls the ratio of promoters in the AP+BP to UP state (Eq 1). In addition,

$$\frac{\left[AP\right]}{\left[BP\right]}=\frac{PPRR}{PBR}+\frac{BTR}{PBR}$$ Eq 2

indicating that the relative magnitude of PBR to BTR+PPRR controls the ratio of promoters in the AP to BP state (Eq 2). As seen in Eq 2, the relative strengths of both PPRR to PBR and BTR to PBR toggle the amount of AP state compared to the BP state. Within our chosen parameter space, PPRR is usually greater than BTR (reflecting the different time scales of these biological processes), allowing the ratio of AP states to BP states to simplify to the relative magnitude of PPRR to PBR. For fixed values of BIR and PBR, lower PPRR and higher BTR values lead to low steady-state RNA production (S1D Fig), consistent with the finding that UP dominates the fractional state probabilities (S1E Fig). With information regarding BIR:BTR and PPRR:PBR ratios, we can summarize the initial fractional distributions of the three promoter states, with the BIR:BTR ratio controlling promoter accessibility (i.e., the amount of closed promoter relative to open promoter, UP to AP+BP), and the PPRR:PBR ratio controlling transcriptional cycling (i.e., the switching between bound and unbound open promoter, AP to BP).

Under steady-state conditions, the fractional distribution of the initial promoter states affects the amount of mRNA produced. We visualized how the promoter accessibility ratio (i.e. BIR:BTR) and the transcriptional cycling ratio (i.e. PPRR:PBR) differentially affect steady-state mRNA production using different heatmap configurations. We first examined the effect of the promoter accessibility ratio (Fig 1B) by varying BIR and BTR (inner heatmaps) for a range of fixed values of PBR and PPRR (outer grid). As PBR and PPRR increased, average steady-state mRNA levels increased, with the highest levels at the bottom right corner (Fig 1B). Within each square (i.e., for a fixed value of PBR and PPRR), average mRNA levels varied with the promoter accessibility ratio, as can be observed by the patterning along the diagonal, with average mRNA increasing from low promoter accessibility ratios (bottom left corner) to high promoter accessibility ratios (upper right corner) within each inner heatmap. In our transcriptional cycling ratio heatmaps for fixed values of BIR and BTR (Fig 1C), we observed a different pattern, noting that average mRNA level increased as PBR and PPRR increased (Fig 1C, upper left to bottom right of each heatmap). However, the smaller relative value of PBR versus PPRR acts as a rate-regulation step for the overall transcriptional cycle [7]. Thus, as BIR increases and BTR decreases across the entire grid, average steady-state mRNA levels generally increase, but the variation is limited by the transcriptional cycling ratio (Fig 1C).

To observe the distribution of initial promoter states varied with these ratios, we zoomed in on one inner heat map. First, we fixed PPRR and PBR at values of 10 (i.e., “outer” PPRR:PBR = 1), and then visually explored how the distribution of initial promoter states varied for a range of promoter accessibility ratios (Fig 1B, box). For this range, we observed that the fraction of promoters that exist in the UP state decreased with increasing accessibility ratio (Fig 1D), consistent with Eq 1 and average mRNA level (Fig 1B). Note that because the transcriptional cycling ratio is 1, the non-UP fraction is equally distributed between the AP (teal) and BP (yellow) states. Repeating this analysis for a fixed promoter accessibility ratio of 1 (Fig 1C, box), we observed that varying the transcriptional cycling ratio changed the relative amounts of AP and BP, while the total fraction of UP remains approximately constant (at ~50% for BIR:BTR = 1), consistent with Eq 2 (Fig 1E). We observed the highest fraction of BP in the bottom left corner, and the highest fraction of AP in the top right corner.

Previous studies suggest that variations in chromatin environments generate cellular heterogeneity [17,25,50,51]. Varied chromatin environments can be represented in the transcriptional cycling model by initializing promoters with different promoter accessibility and transcriptional cycling ratios leading to variations in the distribution of initial promoter states, and therefore we were interested in how varying these ratios would affect transcriptional noise. To examine this, we calculated how the Fano factor (*F* = *σ*^2^/*μ*, where *σ*^2^ is the variance and *μ* is the mean) varied for the same “zoomed in” heatmaps. We observed that the promoter accessibility ratio had a modest influence on the Fano factor, with the highest values generally observed for promoter accessibility ratios < 1 (Fig 1F). Notably, a promoter accessibility ratio < 1 generally characterizes bursty transcription processes, for which activation rates are less than inactivation rates [52]. However, for promoter accessibility ratios << 1, the Fano factor decreases because the fractional probability of non-UP becomes negligible, and very few cells produce transcripts (Fig 1F, bottom left corner).

Interestingly, we observed that noise is independent of the transcriptional cycling ratio, as the Fano factor graph is symmetrical about the diagonal (Fig 1G). Rather, the Fano factor increases as both PPRR and PBR increase because, while the probability of occupying the UP state stays approximately constant, the promoters switching between the AP or BP states produces more mRNA through increased transcriptional cycling, enhancing differences between the closed (UP) and open (AP+BP) states. The rate-limiting nature of the transcriptional cycle in the three-state promoter system requires equal contribution of PBR and PPRR for high levels of transcriptional activity in the basal state. In other words, if only one rate of this cycle increases (for instance, a higher PBR compared to a PPRR), the cycle remains paused. Thus, while PPRR and PBR control amounts of AP and BP respectively, changes in expression and noise are more dependent on the absolute values of PPRR and PBR.

### Activating gene expression under different promoter initialization states affects transcriptional noise and cell-to-cell heterogeneity

A key biological question is how activation of TFs upon exogenous stimulation affects transcriptional noise [27,28]. TFs often recruit molecular complexes that alter one or more of the rates in the transcriptional cycling model. For example, as discussed above, a consequence of activating NF-κB is to mediate the recruitment of P-TEFb, releasing paused RNAPII and allowing transcriptional elongation to proceed more efficiently [32–34]. Because regulation of RNAPII pause release is a widely conserved mechanism to control transcription [53–56], we simulated this activation pathway by increasing the pause release rate, PPRR, in our model and quantified how it affected transcriptional noise.

We first conducted a sensitivity analysis of how increasing PPRR affected transcription over 24 hours, monitoring mRNA over various combinations of BIR, BTR and PBR. For this and the following studies of inducible transcription, we limited our parameter range to match those previously reported in the transcriptional bursting literature (Table 1). The four parameters were varied over two orders of magnitude, which enabled exploration of a wide range of initial fractional promoter states. We selected a 2-fold increase in PPRR as an appropriate activation signal (S2A Fig), as this increased gene expression over basal conditions (mRNA > 3) without excessive activation. Starting from the basal steady-state (i.e., the state after 10-day simulations with basal PPRR), we tracked activation of transcription by running 1,000 stochastic simulations of the model for each parameter condition for 24 hours following a 2-fold increase in PPRR.

**Table 1:** Model development and parameter space.

Reaction	Rate	Value / Range	References
*UP*→*AP*	BIR (Burst Initiation Rate)	[0.01–1] hr^-1^	[7,52,57–60]
*AP*→*BP*	PBR (Polymerase Binding Rate)	[1–100] hr^-1^	[7,52,57,61–63]
*AP*→*UP* *BP*→*UP*	BTR (Burst Termination Rate)	[0.01–1] hr^-1^	[7,52,57,58,60]
*BP*→*RNA*+*AP*	PPRR (Polymerase Pause Release Rate)	[1–100] hr^-1^	[7,18,52,62,63]
*RNA*→*Protein*	Kp (Translation Rate)	1	[4,48]
*RNA*→∅	DegR (Degradation of RNA Rate)	0.34	[4,18]
*Protein*→∅	DegP (Degradation of Protein Rate)	0.1	[4,49]

**Fig 2:**
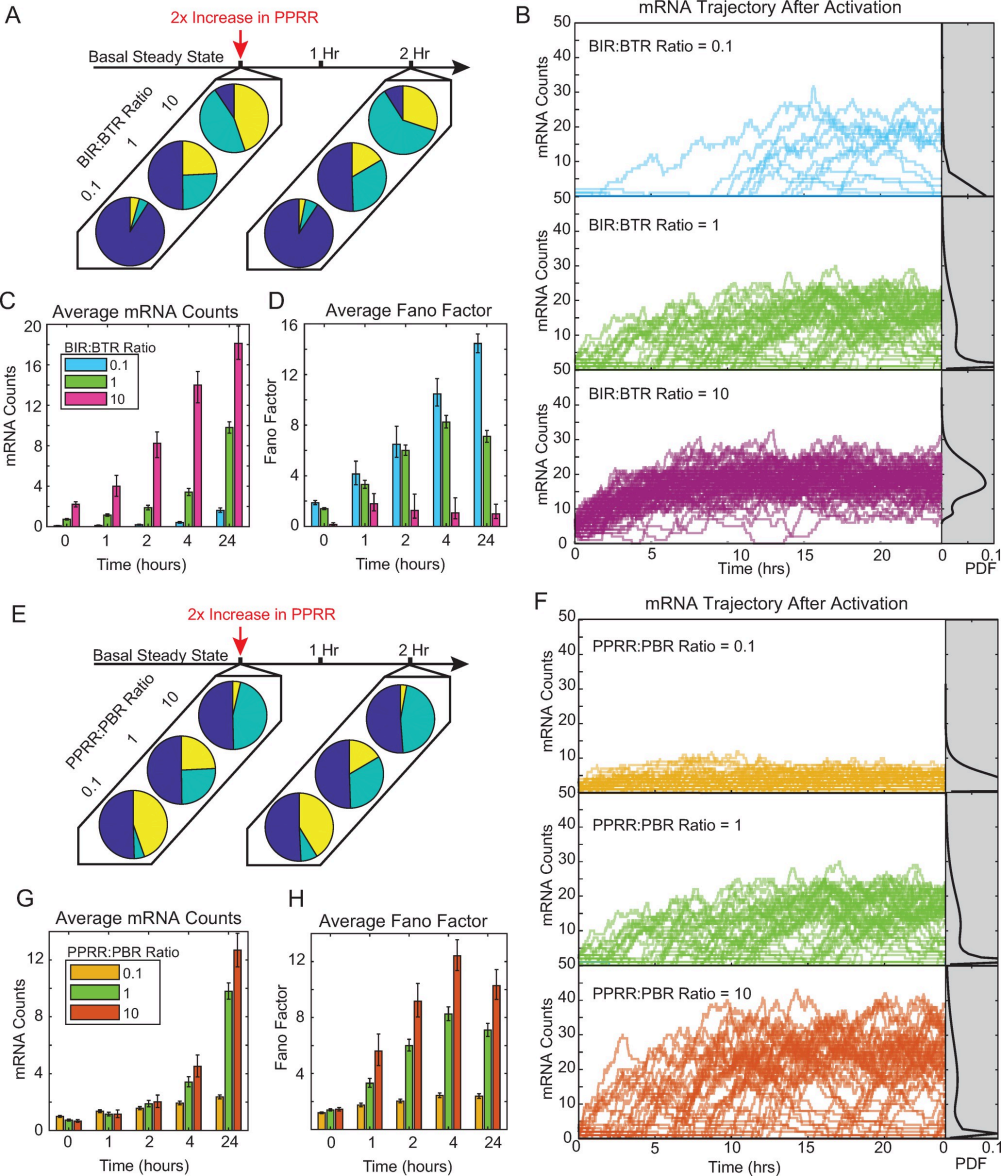
The initial distribution of promoter states influences the heterogeneity in transcriptional activation modeled as an increase in PPRR. (A) Three representative pie charts of fractional promoter-state probability of UP (blue), AP (teal) and BP (yellow) for BIR:BTR ratios of 0.1, 1, and 10 with PBR and PPRR held constant at 10 hr^-1^. Basal conditions were calculated from 1000 single-cell stochastic simulations out to 10 days for each parameter combination. At time = 0, PPRR was increased two-fold (B), and new fractional probabilities were captured at 2 hours. (B) Representative trajectories for BIR:BTR = 10 (pink), BIR:BTR = 1 (green), and BIR:BTR = 0.1 (cerulean). Each line represents one stochastic simulation out to 24 hours. Only 100 simulations are plotted for each condition for ease of visualization. Gray regions on the right represent the probability density of mRNA counts at 24 hours, with kernel smoothing. (C-D) Average mRNA counts (C) and Fano factor (D) for the three BIR:BTR ratios at 0, 1, 2, 4 and 24 hours. Average mRNA values and Fano factor were calculated from 1,000 single-cell stochastic simulation for each parameter combination. Error bars represent 95% bootstrapped confidence intervals. (E) Three representative pie charts of fractional promoter-state probability of UP (blue), AP (teal) and BP (yellow) for PPRR:PBR ratios of 0.1, 1, and 10 with BIR and BTR held constant at 0.1 hr^-1^. Fractional probabilities were calculated as described in (A). (F) Representative trajectories for PPRR:BBR = 0.1 (yellow), PPRR:PBR = 1 (green), and PPRR:PBR = 10 (orange). Data presented as described in (B). (G-H) Average mRNA counts (G) and Fano factor (H) for the three PPRR:PBR ratios at 0, 1, 2, 4 and 24 hours. Data presented as described in (C-D).

We first examined activation trajectories for promoters initiated with three different promoter accessibility ratios (0.1, 1 and 10) for the same fixed PBR and PPRR as in Fig 1B. Increasing the promoter accessibility ratio increased the fraction of promoters in the AP+BP states relative to the UP under basal conditions (Fig 2A, left). Upon activation (i.e., a 2-fold increase in PPRR), a fraction of promoter states switched from the BP to AP state, but the fraction of promoters in the UP state remained approximately constant for all three ratios, as increasing PPRR does not affect the UP state (Fig 2A, right). Examining the transcriptional trajectories over 24 hours revealed three distinct behaviors (Fig 2B). Initializing promoters with a low promoter accessibility ratio (BIR:BTR = 0.1) rarely led to transcriptional activation, as evidenced by only a few non-zero trajectories and a probability density function (PDF) containing cells that are mostly off (Fig 2B, top). In contrast, initializing promoters with a high promoter accessibility ratio (BIR:BTR = 10) resulted in almost complete activation (Fig 2B, bottom). Interestingly, initializing with a promoter accessibility ratio of 1 resulted in heterogeneous outcomes, characterized by bimodal activation. As expected, average mRNA increased over 24 hours in all cases, with the largest absolute increase observed for the promoter accessibility ratio of 10 (Fig 2C). Transcriptional noise (as quantified by the Fano factor) also increased over 24 hours in all cases, although promoter accessibility ratios of 10 and 1 peaked at 1 and 4 hours respectively (Fig 2D). This plateau of noisy expression for the higher promoter accessibility ratios reflect the time required for the stimulated system to equilibrate after PPRR increases (Fig 2B, bottom and middle panel). Overall, this analysis illustrates how different distributions of initial promoter states lead to varied phenotypes upon activation, with the basal fraction of UP inversely correlated to the fraction of activating cells.

Next, we examined how varying the transcriptional cycling ratio (0.1, 1, and 10) contributes to noise and heterogeneity upon PPRR activation. As noted previously, under basal conditions, as the ratio of transcriptional cycling decreases, the fraction of BP increases, while the fraction of UP remains constant (Fig 2E, left). Upon stimulation (i.e., a 2-fold increase in PPRR at time = 0), the amount of AP increased marginally by 2 hours (Fig 2E, right). Increasing the transcriptional cycling ratio over 24 hours increased both the fraction of promoters actively transcribing (Fig 2F) and the average mRNA by 24 hours (Fig 2G). This increase in mRNA production is accompanied by increases in noise (Fano factor; Fig 2H). This increase peaked around the 4-hour mark for transcriptional cycling ratios of 1 and 10, corresponding to when trajectories begin to plateau, and leads to bimodal mRNA levels across cells by 24 hours (Fig 2F, middle and bottom grey). Lower transcriptional cycling ratios lead to lower levels of gene transcription, and decreased Fano factor, as the trajectories cluster near low expression levels (Fig 2F, top). However, while higher transcriptional cycling ratios increased activation, noise also increased, because the UP fraction remained unchanged and thus there were non-activated cells by 24 hours even at the highest transcriptional cycling ratio. Notably, it is possible that the transcriptional cycling ratio might saturate, i.e. due to local depletion of p-TEFb which would limit the rate of polymerase pause release [64], in which case we would expect to reach an upper limit on mRNA expression count and noise, after which they would start to decrease.

To generalize our observations, we classified sections of the parameter space as “always on,” “always off,” and “bimodal” (for classification breakdown, see Materials and Methods) (S2B Fig). We then analyzed the initial fractional promoter states for each of these phenotypes (S2C Fig). The “always off” phenotype had much higher levels of UP relative to AP and BP, whereas the “always on” phenotype had approximately equivalent amounts of each promoter. The “bimodal” phenotype was in the middle, with higher UP as compared to the “always on” population, but lower BP and AP as compared the “always off.” This suggests that the distribution of initial promoter states contributes to bimodality following transcriptional activation.

### Positive feedback that amplifies the polymerase pause release rate (PPRR) in the transcriptional cycling model does not affect bimodality

Positive feedback is a common motif used to activate and amplify gene expression [38,49], and so we next sought to explore how positive feedback would influence gene expression noise following inducible activation. There are many different biological mechanisms of positive feedback, and so we limited our exploration to the mechanism used by HIV. Briefly, upon initiation of HIV transcription, the initiated transcripts form a stem-loop structure referred to as the transactivating response region (TAR), which leads to promoter proximal pausing by RNAPII [65]. HIV encodes its own transcriptional transactivator (Tat), which recruits P-TEFb to the transcriptional start site [66,67] where it releases paused RNAPII to enable elongation and the generation of a full-length transcript [6,36,68]. HIV exhibits bimodal activation under basal conditions [69] and following stimulation with TNF [70], and therefore we were interested to see to what extent positive feedback contributed to this observation.

**Fig 3:**
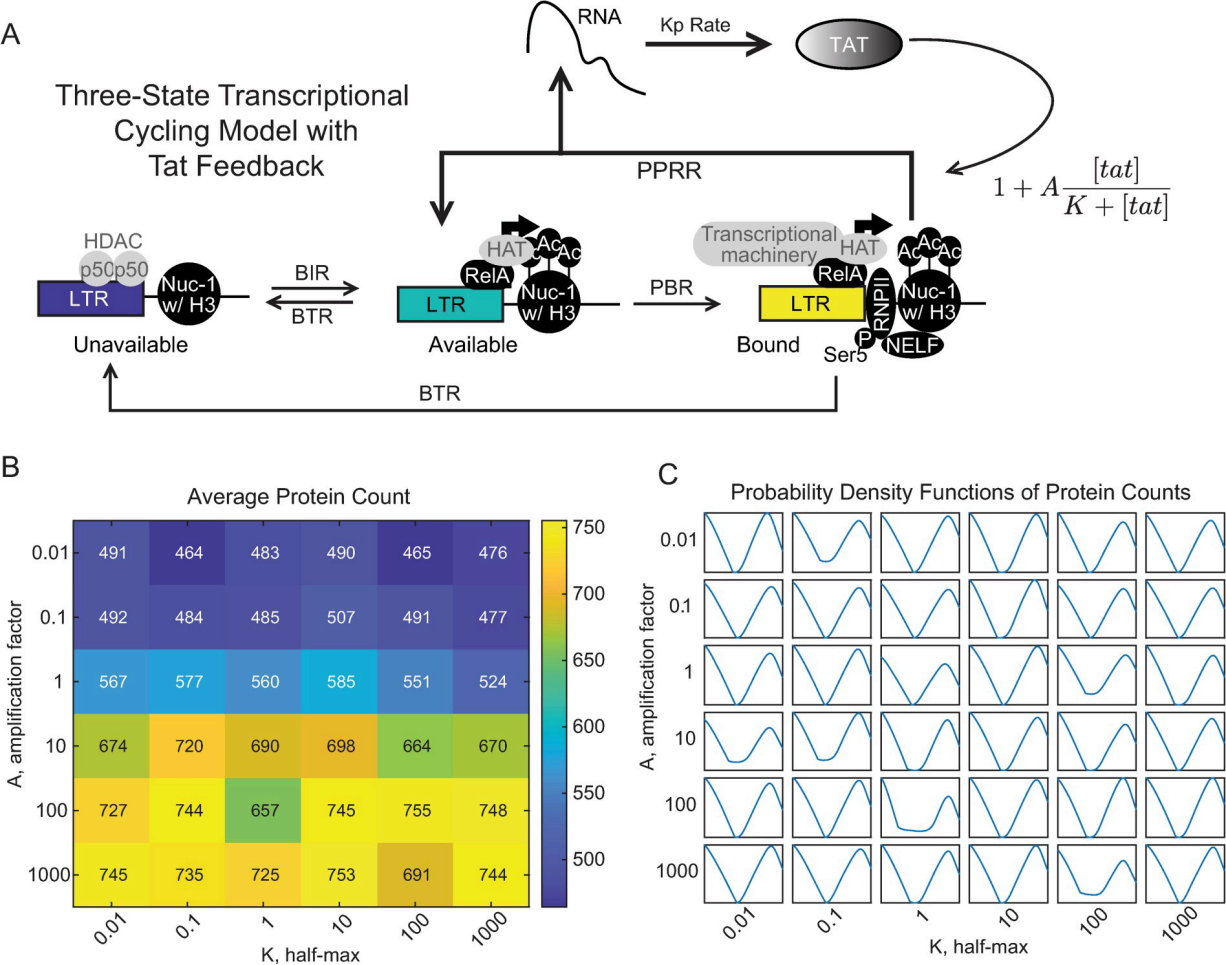
Positive feedback on PPRR activation does not influence bimodality of the mRNA and protein distributions in the three-state transcriptional cycling model. (A) Updated three-state promoter system with HIV nucleosome remodeling, RelA recruitment, and Tat-mediated transcript elongation, which is amplified via positive feedback. Positive feedback is modeled as a saturating function with an amplitude, A, and half-max, K. (B) Heatmap of average protein counts at 24 hours with feedback. Protein counts were generated through stochastic simulation for 1,000 cells for each combination of K and A, which were varied over 5 orders of magnitude. The other parameters were fixed as follows: BIR = 0.1 hr^-1^, BTR = 1 hr^-1^, PBR = PPRR = 10 hr^-1^ (C) Kernel fittings of mRNA counts at 24 hours. Each box contains the probability density curve for that parameter combination.

We added Tat-mediated positive feedback to the transcriptional cycling model by amplifying PPRR with a Tat-dependent term (Fig 3A) as shown:

$$Feedback=PPRR*\left(1+A*\frac{\left[TAT\right]}{K+[TAT]}\right)$$ Eq 3

in which K determines the half-maximal saturation and A is the amplitude of the feedback. We performed a sensitivity analysis to determine how these two terms affected transcriptional activation by 24 hours. We chose a region of the parameter space with bursty characteristics (promoter accessibility ratio < 1), and a transcriptional cycling ratio = 1 to remove any rate limiting effects downstream of promoter transition from unavailable to available. We found that increasing A by five orders of magnitude only increased average mRNA and protein levels approximately 1.5-fold, while increasing K across a similar range had almost no effect (Fig 3B and S3A Fig). Varying A and K did not affect the fraction of promoters in the UP state (S3B Fig), consistent with the analysis that the UP fraction is unaffected by changes in PPRR. Varying A did affect the fraction of promoters in the AP and BP states, while K had no effect. As the amount of AP saturates the fractional availability, the additional feedback strength will not alter transcriptional production. The influence of K on the sensitivity of the feedback can be visualized by plotting the relative feedback value for varying levels of K. As K decreases, it reaches high feedback strength at very low level of protein production, saturating the signal (S3C Fig). Once saturated, the reaction regulation rate becomes PBR and amplifying PPRR via positive feedback will not lead to further increases in protein production.

Previous studies have suggested that Tat-positive feedback is required for bimodality of protein production [71]. However, we observed that, for our regions of interest in the parameter space, implementing positive feedback as an amplification of PPRR did not alter the bimodality observed in the absence of feedback (Fig 3C). Overall, we conclude that for the transcriptional cycling model in which Tat feedback only affects RNAPII pause release (i.e., PPRR), Tat positive feedback increases overall expression but does not alter bimodality.

### Positive feedback on PPRR increases transcriptional noise when initialized from promoter states with high PPRR:PBR ratios

Although Tat-mediated feedback on PPRR in the transcriptional cycling model does not affect bimodality, it does influence the rate of transcriptional cycling, increasing the rate at which BP transitions back to AP with a release of transcript (Fig 3A). To visualize how feedback affects activation in different regions of the parameter space, we returned to our promoter accessibility (BIR:BTR) and transcriptional cycling (PPRR:PBR) ratios to examine mRNA averages, noise profiles, and trajectories after activation. We modeled Tat feedback through the addition of Eq 3, and stochastically simulated our three-state system over an interval of ten days to reach a basal steady-state. The system was then activated by increasing PPRR 2-fold for the same parameter regions explored in Fig 2, but this time in the presence of Tat feedback.

**Fig 4:**
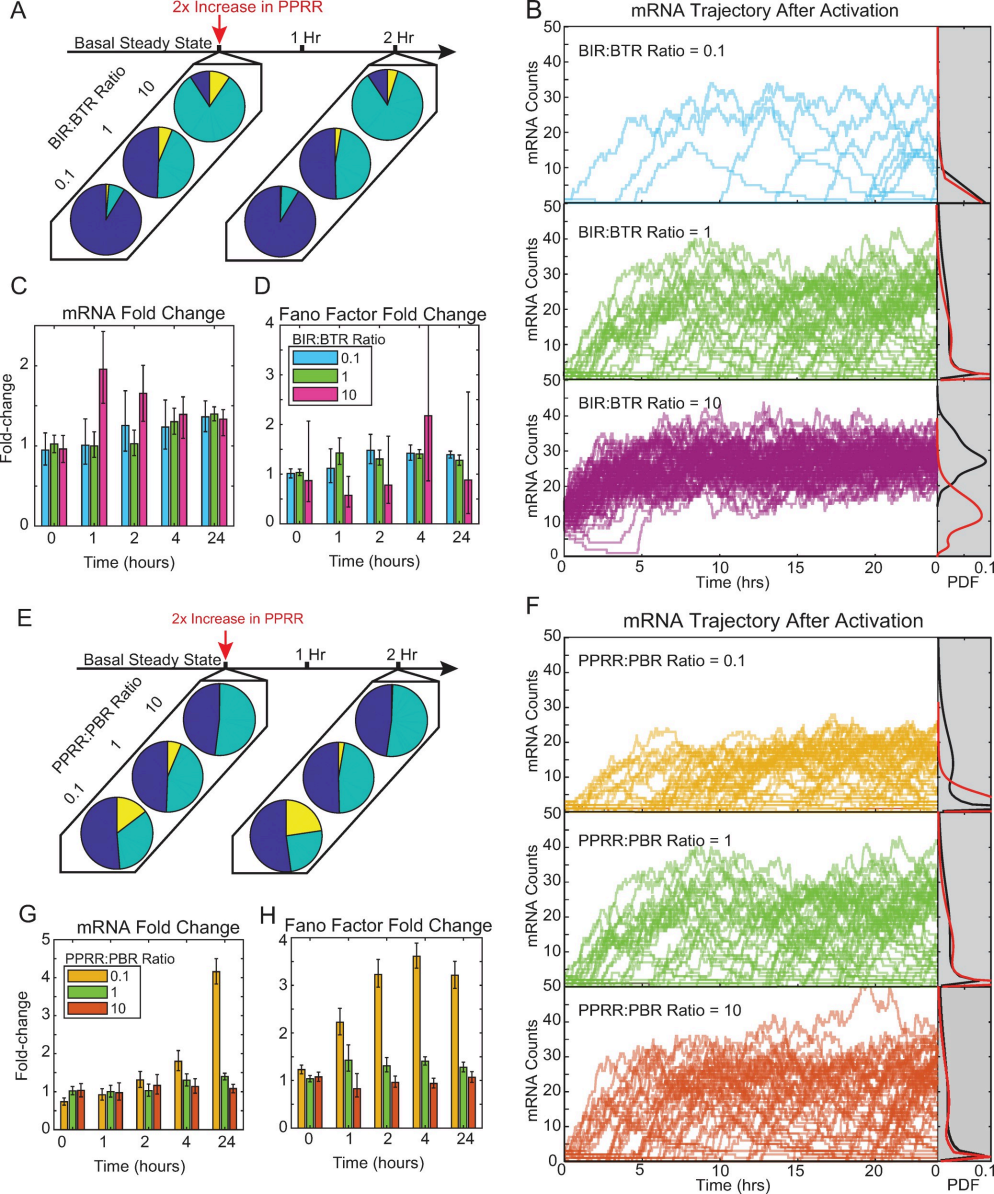
Transcriptional activation in the presence of PPRR positive feedback predominately alters activation of more permissive initial promoter states. (A) Three representative fractional promoter-state probability pie charts with the addition of feedback of UP (blue), AP (teal) and BP (yellow) for BIR:BTR ratios of 0.1, 1, and 10 with PBR and PPRR held constant at 10 hr^-1^. Data presented as described in Fig 2A. (B) Representative simulated trajectories with feedback for BIR:BTR = 10 (pink), BIR:BTR = 1 (green), and BIR:BTR = 0.1 (cerulean) presented as described in (Fig 2B). Gray regions on the right represent the probability density of mRNA counts at 24 hours, with kernel smoothing, with feedback (black) and without feedback (red). (C-D) Fold-change in mRNA counts (C) and Fano factor (D) for the three BIR:BTR ratios at 0, 1, 2, 4 and 24 hours as compared to non-feedback simulations. Data were generated through stochastic simulation for 1,000 cells for each parameter combination. Error bars represent 95% bootstrapped confidence intervals. (E) Three representative pie charts of fractional promoter-state probability with the addition of feedback of UP (blue), AP (teal) and BP (yellow) for PPRR:PBR ratios of 0.1, 1, and 10 with BIR and BTR held constant at 0.1 hr^-1^. Fractional probabilities were calculated as described in (A). (F) Representative simulated trajectories with feedback for PPRR:PBR = 0.1 (yellow), PPRR:PBR = 1 (green), and PPRR:PBR = 10 (orange). Data presented as described in (B). (G-H) Fold-change mRNA counts (G) and Fano factor (H) for the three PPRR:PBR ratios for timepoints of 0, 1, 2, 4 and 24 hours as compared to non-feedback simulations. Data presented as described in (C-D).

We first examined how feedback affects activation for different promoter accessibility ratios. Although feedback does not affect the initial fraction of promoters in the UP state, it does move more of the non-UP fraction from the BP to the AP state (Fig 4A, left), as the effective PPRR is increased with the addition of feedback. After activation via a 2-fold increase in PPRR, even more of the BP fraction is converted to AP (Fig 4A, right). We saw similar trends in the trajectories of the three promoter accessibility ratios as compared to our results without feedback (Fig 4B; compare to Fig 2B), however they were differentially affected by feedback. For the highest promoter accessibility ratio (BIR:BTR = 10), feedback increased average mRNA by approximately 1.4-fold over 24 hours (Fig 4B, top, black versus red, and Fig 4C). By contrast, feedback only moderately increased average mRNA for the intermediate promoter accessibility ratio (BIR:BTR = 1), and it had almost no effect on the lowest ratio (BIR:BTR = 0.1; Fig 4B, middle and bottom), as most of these promoters remained in the UP state and thus were unaffected by PPRR amplification.

Feedback produced only minor increases in noise (i.e., Fano factor), with the lowest promoter accessibility ratio exhibiting the largest increases by 24 hours (Fig 4D). As expected, feedback played a larger role in amplifying gene transcription for cells that had higher promoter accessibility ratios (and thus less UP). Feedback amplified a few highly active trajectories for lowest promoter accessibility ratio, but did not alter simulations that were initialized in the UP state, leading to an overall increase in the Fano factor.

Feedback also variably affected activation trajectories initialized across different transcriptional cycling ratios (0.1, 1, and 10; Fig 4E). For the highest ratio (PPRR:PBR = 10), increasing PPRR via feedback did not significantly change the distribution of trajectories, average mRNA levels, or noise over time (Fig 4F–4H), because PBR was already the regulating reaction for transcription for this ratio. For the two lower ratios (0.1 and 1), feedback increased both mRNA production and noise over 24 hours (Fig 4G–4H). Examining the trajectories, we qualitatively observed a few outlier cells that exhibited large increases in mRNA production with feedback, which likely correspond to the small fraction of cells initialized in the BP state, because feedback amplifies the signal of cells that have available BP. For parameter sets that lead to few cells initiated in the BP state, feedback on PPRR does little to change the phenotype of stimulated cells.

### The three-state transcriptional cycling model qualitatively reproduces experimentally observed activation of latent HIV

**Fig 5:**
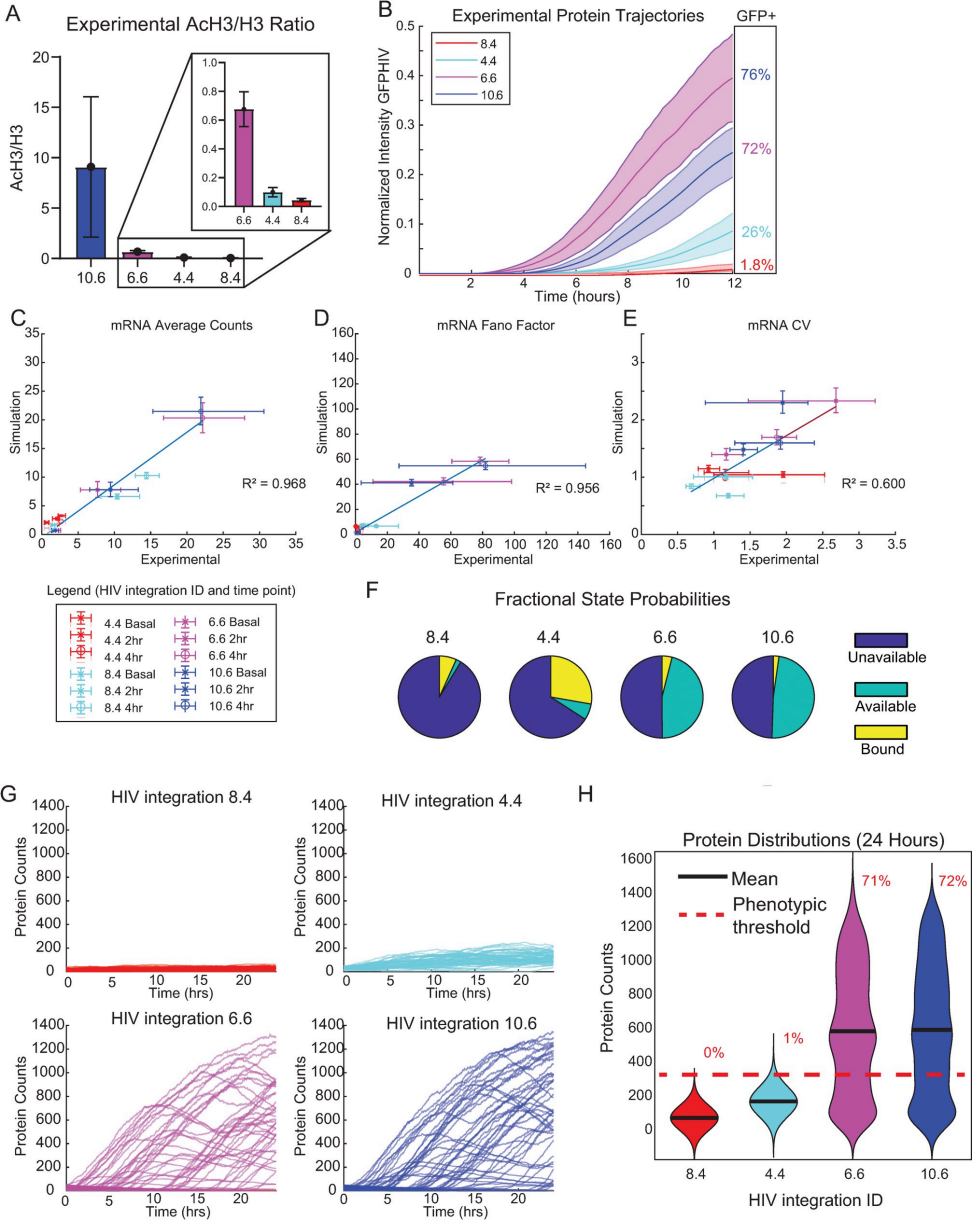
The three-state transcriptional cycling model reproduces transcriptional activation heterogeneity observed for a range of latent HIV integrations. (A) Ratio of enrichment of total histone H3 to acetylated H3 (AcH3) in Jurkat T cells at the indicated target promoters quantified by ChIP-qPCR. Data are presented as mean of % input (non-IP control) ± SD of two biological replicates. (B) Experimental GFP-HIV trajectories for the four HIV integrations, plotted with 95% confidence intervals and normalized via experimental setup. (C-E) Scatterplots of three-state promoter simulation with feedback compared to experimental measurements for mRNA Average (C), Fano factor (D), and CV (E). Error bars represent 95% bootstrapped confidence intervals (C) Fractional state probabilities under basal conditions of UP (blue), AP (teal) and BP (yellow) for the four integrations based upon the three-state model. Simulations were run 10,000 times. (F) Representative simulated protein trajectories over 24 hours of the 4 integrations. (G) Violin kernel fitting of protein distributions at basal conditions and at 24 hours of the four integrations. Black bar represents mean. Red dashed line represents a protein threshold of 275 with percentages as the amount above that threshold. Experimental data in this figure reproduced from [4].

We next explored how accurately the transcriptional cycling model could reproduce experimental data on latent HIV activation. We and others have previously used a random telegraph model to describe HIV bursting dynamics [5,18,45,49]. However, the random telegraph model with only two promoter states did not fully the differences in chromatin environment that affected transcriptional bursting of latent viral integrations. For example, for LTR integrations that exhibit similar transcriptional bursting dynamics in the basal state (i.e., are fit by the same basal parameters in the two-state model and have average counts of basal mRNA < 3), we can measure significant differences in the local chromatin environment, as evaluated by the ratio of acetylated histone 3 to total histone 3 (AcH3:H3, Fig 5A). These differences in the basal state are associated with significant differences in transcriptional activation upon stimulation with TNF [4] (Fig 5B). This suggests that the two promoter states of the random telegraph model are insufficient to describe the transcriptional dynamics of these latent HIV integrations in the basal state and during TNF-mediated transcriptional activation. Moreover, stimulation of viral activation using the two-state model did not recapitulate bimodality, a known feature of HIV expression [69,70]. Therefore, we sought to determine if the transcriptional cycling model could better reflect the biology of HIV reactivation.

To determine optimal parameter sets describing the four latent HIV integrations, we fit our model to experimental measurements of transcript distributions at 0-, 2- and 4-hours post TNF treatment in four clonal Jurkat T cell populations each with one of the viral integrations [4], identified here as Integration 8.4, Integration 4.4, Integration 6.6, and Integration 10.6. As described above, TNF activation was simulated by increasing PPRR 2-fold. Using a selection algorithm (see Materials and Methods), we found four sets of parameters that minimized error across three features of the experimental measurements of mRNA distributions: the average transcript level, Fano factor, and coefficient of variation (CV) (S4A–S4C Fig; Table 2).

**Table 2:** Parameters selected for experimental fitting with PPRR activation.

Parameter	Fittings for Integration 8.4	Fittings for Integration 4.4	Fittings for Integration 6.6	Fittings for Integration 10.6
BIR	1 hr^-1^	0.5 hr^-1^	0.1 hr^-1^	0.1 hr^-1^
BTR	10 hr^-1^	1 hr^-1^	0.1 hr^-1^	0.1 hr^-1^
PBR	50 hr^-1^	50 hr^-1^	50 hr^-1^	50 hr^-1^
PPRR	1 hr^-1^	1 hr^-1^	50 hr^-1^	100 hr^-1^

We then added Tat positive feedback into the transcriptional cycling model and compared simulation to experimental measurements of transcript distributions in the presence of feedback without further fitting (Fig 5C–5E). We found that average mRNA, Fano factor, and CV calculated from 1000 simulations reproduced our experimental measurements with feedback with good accuracy (R^2^ values of 0.968, 0.956, and 0.60 respectively), demonstrating that the chosen feedback parameters can reproduce Tat amplification.

When we analyzed the initial promoter states for the selected parameter set for the four integrations, we noted that viral integrations with higher ratios of AcH3:H3 were fit with parameters that exhibited higher ratios of (AP+BP):UP (Fig 5F) but that all were classified as latent (i.e., exhibit average counts of basal mRNA levels < 3). Thus, with the additional parameters to account for chromatin remodeling and polymerase recruitment and release in the three-state model, we identified distributions of initial promoter states that more accurately reflected experimentally measured differences in the chromatin environment.

Finally, we compared our simulations of TNF-activated HIV Tat protein expression over 24 hours to our experimental measurements [4]. We found that increasing PPRR by 2-fold across all four integrations produced significant differences in viral activation by 24 hours. Integrations 6.6 and 10.6 exhibited high levels of activation (calculated as 71% and 72% respectively above a basal threshold of 275 simulated proteins, shown via the red dashed line), while 4.4 and 8.4 were not strongly activated (Fig 5G). Moreover, activation of 6.6 and 10.6 was bimodal (Fig 5G), as we had observed experimentally by flow cytometry. While the transcriptional cycling model more accurately captured the chromatin states underlying HIV activation differences, a 2-fold increase in PPRR alone did not fully replicate reactivation seen experimentally. Also, the activation of 4.4 did not produce the bimodality seen experimentally. To address these issues, we turned to a multi-step activation model.

### Activation of the transcriptional cycling model via multiple paths reproduces additional observed features of latent HIV activation

**Fig 6:**
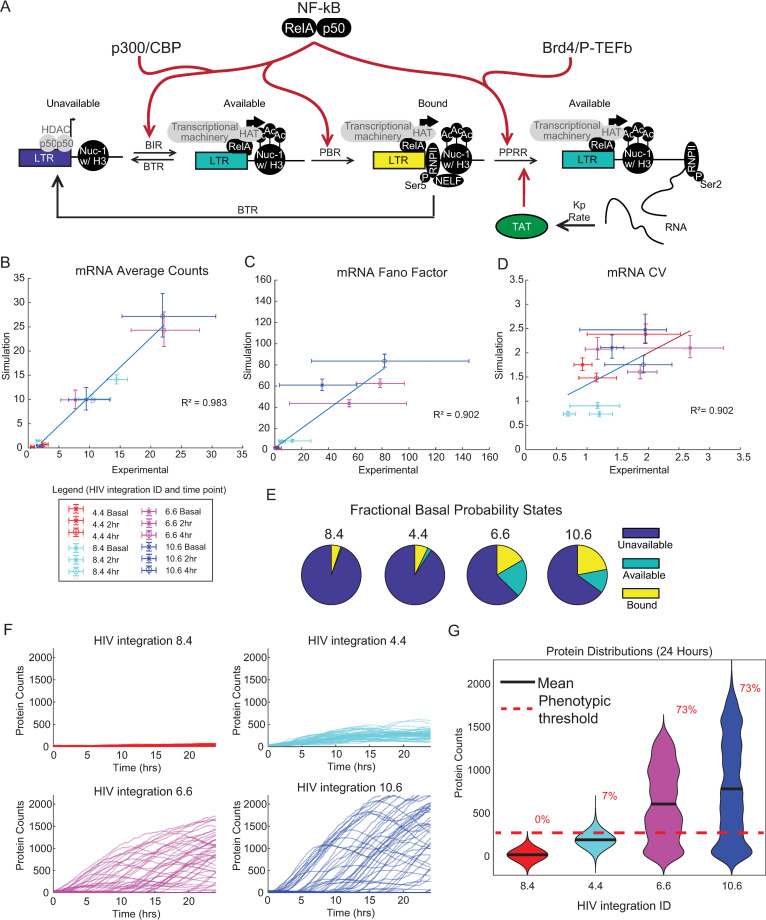
Implementing transcriptional activation via increases in multiple parameters reproduces transcriptional activation heterogeneity observed for a range of latent HIV integrations with more biological accuracy. (A) Schematic of multi-point three-pronged activation in the transcriptional cycling model for HIV. (B-D) Scatterplots of three-state promoter simulation with feedback compared to experimental measurements for mRNA Average (B), Fano factor (C), and CV (D). Error bars represent 95% bootstrapped confidence intervals. (E) Fractional state probabilities under basal conditions for the four integrations based upon the three-state model. Simulations were run 1,000 times. (F) Simulated trajectory protein data of 50 representative cells out to 24 hours of the four integrations. (G) Violin kernel fitting of protein distributions at basal conditions and at 24 hours of the four integrations. Black bar represents the mean. Red dashed line represents a protein threshold of 275 with percentages as the amount above that threshold.

As described above, following stimulation by the inflammatory TNF, NF-κB mediates steps in transcriptional activation in addition to its role in the recruitment of P-TEFb [35]. NF-κB also mediates recruitment of CBP/p300 for chromatin remodeling [30], as well as Mediator, RNAPII and other members of the preinitiation complex [31]. Just as we modeled the role of NF-κB in recruiting P-TEFb (i.e., through a 2-fold increase in PPRR), we can recapitulate these additional steps in regulating promoter accessibility and RNAPII binding as fold increases in BIR and PBR, respectively (Fig 6A).

To capture the multi-factorial role of NF-κB activity following TNF stimulation, we increased BIR, PBR, and PPRR 2-fold from basal state conditions across a range of initial parameter values that were consistent with the transcriptional profiles of latent integrations (i.e., average count of mRNA < 3). Then, using a similar methodology as our PPRR-only activation scheme, we selected a parameter space that most optimally fit the features of transcription at 0, 2 and 4 hours for the experimental measurements of the four latent integrations without feedback (S5A–S5C Fig). We then ran simulations in the presence of feedback to predict transcript count and distribution at basal, 2, and 4 hours. The parameters chosen as predictions are shown below in Table 3.

**Table 3:** Parameters selected for experimental fitting with multi-point activation.

Parameter	Fitting for Integration 8.4	Fitting for Integration 4.4	Fitting for Integration 6.6	Fitting for Integration 10.6
BIR	0.5 hr^-1^	0.5 hr^-1^	0.05 hr^-1^	0.05 hr^-1^
BTR	10 hr^-1^	5 hr^-1^	0.1 hr^-1^	0.1 hr^-1^
PBR	100 hr^-1^	100 hr^-1^	50 hr^-1^	100 hr^-1^
PPRR	1 hr^-1^	5 hr^-1^	10 hr^-1^	10 hr^-1^

These predictions for mRNA average, CV, and Fano factor showed good fit with our experimental dataset in the presence of feedback with an *R*^2^>0.90 (Fig 6B–6D). The fractional distribution of initial states found to be optimal for these integration positions under basal and latent conditions changed when activation increased all three parameters. All viral integrations had higher levels of UP as compared to the previous simulation (Fig 6E), which is consistent with an activating transcription factor that can act on closed, unavailable chromatin, as NF-κB is known to do. In addition, these parameter sets better capture bursty parameter spaces, where BIR < BTR for all four integrations (Table 2). Jurkat cells harboring viral integrations 6.6 and 10.6 again exhibited bimodal protein distributions by 24 hours (Fig 6F–6G), with 73% of the population activated above threshold. Integration 4.4 activated to 7% above threshold, suggesting that this multi-activation model better captures reactivation for these more repressed viral integrations. Further optimization might be achieved through reparameterization of feedback terms or fine-tuning of fold-change activations. Altogether, these results demonstrate how implementing multiple TF activation paths in our three-state model more accurately replicates NF-κB-mediated activation of latent HIV viruses.

### The upstream activation path of the transcriptional cycling model differentially affects features of noisy inducible transcription

To compare how each ‘activation path’ contributed to multi-step activation, we took the fitted parameter combinations for the four viral integrations, and examined how BIR, PBR, and PPRR fold-change activation alone affected noise as compared to a multi-step activation. While this parameter space may not replicate our observed experimental data, it allowed for direct comparison across low and high activating viral integrations. For every activation pathway, BIR, PBR, PPRR, or Multi (activating BIR, PBR, and PPRR simultaneously), the appropriate pathway parameter(s) were increased 2-fold and simulated for 24 hours.

Examining transcripts and protein counts at basal and 24 hours post activation (Figs S6A and 7A), we see similar patterns across all viral integrations, with the Multi pathway producing the most transcript and proteins out to 24 hours whatever the initial starting state. However, noise (as quantified by Fano factor) is highest for the low-activating 8.4 and 4.4 viral integrations when activated via the PPRR pathway, while it is highest for 6.6 and 10.6 when activated via the PBR pathway (Figs S6B and 7B). Notably, activation via the BIR pathway leads to a marked decrease in Fano factor because it increases the frequency of switching between the open and closed promoter states, leading to less separation between the productive and unproductive populations.

**Fig 7:**
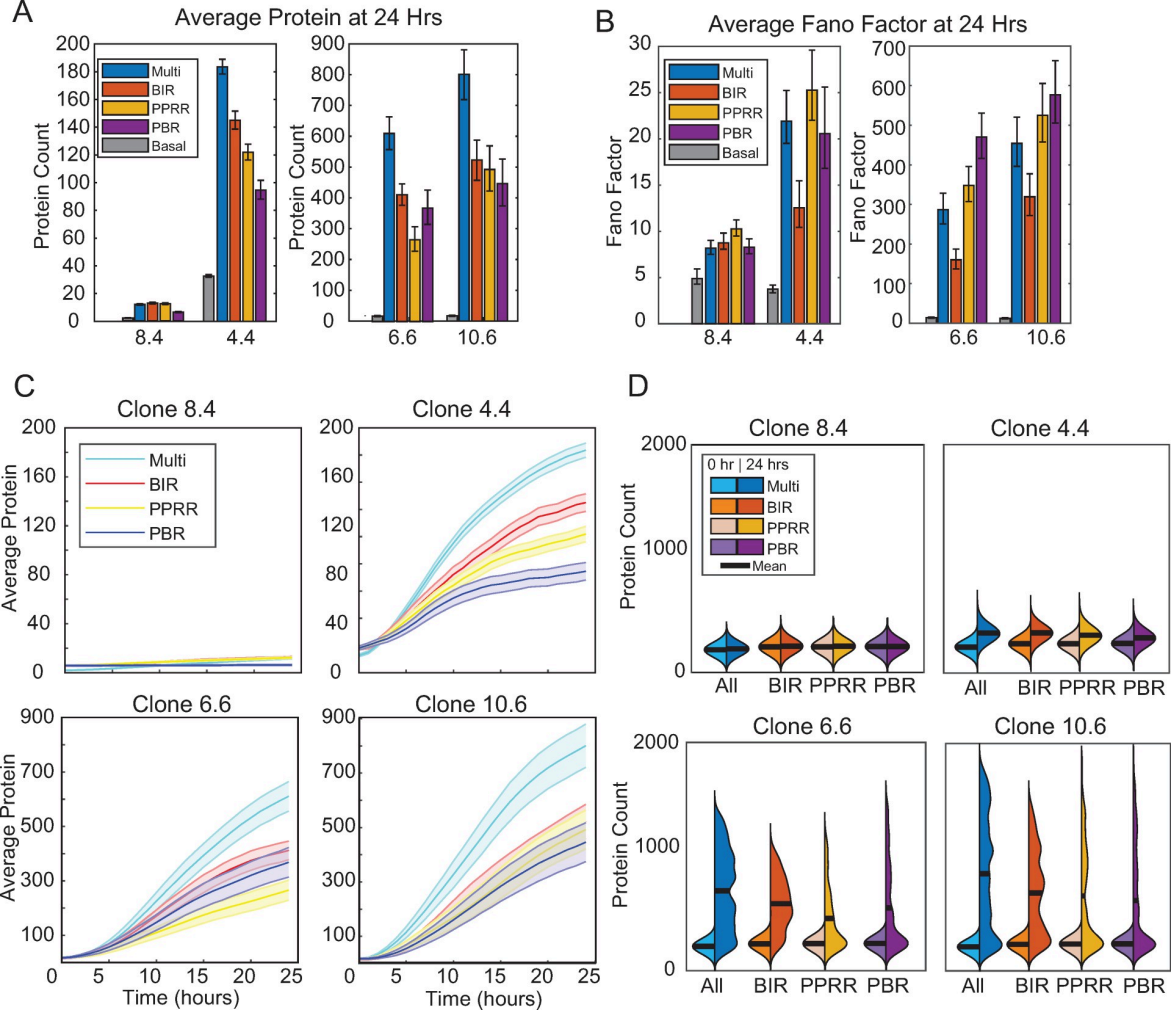
Multi-step activation can be broken down into discrete activation lines which influence noise and protein counts by 24 hours. (A-B) Average protein counts (A) and Fano factor (B) for the four activation options at 24 hours. Protein counts were generated through stochastic simulation for 1,000 cells for each parameter combination. Error bars represent 95% bootstrapped confidence intervals. (C) Simulated trajectory protein data of 50 representative cells out to 24 hours of the four activation lines with the four integrations. (D) Violin kernel fitting of protein distributions at basal conditions and at 24 hours of the four activation lines with the four integrations. Black bar represents the mean.

To visualize these differences in noise, we plotted the averaged protein trajectories along with kernel probability density functions for each activation pathway (Fig 7C and 7D). The variations across trajectories for each viral integration highlighted the basal configuration (Fig 6E) upon activation, with 4.4’s changes in BIR leading to increases in simulated protein (Fig 7C top right), as this promotes UP to AP+BP changes. For 6.6 and 10.6, the multi-point activation overall saw the highest increases, with no clear delineation among the individual activation lines. We also compared final protein counts at 24 hours as compared to basal conditions (Fig 7D). Increases to PPRR and PBR for 6.6 and 10.6 lead to more skewed distributions (Fig 7D bottom), with the highest noise but lower activation (S6C Fig). From these comparisons, we can conclude that BIR activation leads to lower noise across integration sites, without substantial changes to protein average. In contrast, PPRR and PBR increase noise through a few highly productive cells, leading to more skewed distributions. These findings of how model reactions rates relate to gene expression noise are consistent with analytical solutions previously solved and reported for the steady-state three-state transcriptional cycling model [7]. Overall, we conclude that phenotypic heterogeneity following activation by exogenous stimulation depends on the distribution of the initial promoter states, as well as the biological activation pathway.

## Discussion

Despite widespread observations of transcriptional bursting, the molecular mechanisms regulating bursting remain unclear. While experimental studies have suggested a variety of contributors, including RNAPII pausing and chromatin remodeling, the canonical mathematical model of transcriptional bursting is too simple to explore these mechanisms using simulations, and it restricts integrating models of transcriptional bursting with specific processes affected by upstream signals and TFs.

Here, we used a three-state transcriptional cycling model [19] to explore how chromatin variations, polymerase initiation and pausing contribute to transcriptional bursting. Previous work has highlighted the importance of burst initiation and polymerase pause release on control of transcription through this model [7,19]. We found that the variation in the fractional promoter state probabilities produced by this three-state transcriptional cycling model more accurately described the range of promoter configurations generated by epigenetic variations at basal state. The motivation for this work was to represent the varied chromatin environments at NF-κB-inducible promoters, including the HIV LTR, which lead to altered transcriptional expression and noise, as we highlighted in recent work [4,28]. Indeed, we found that the transcriptional cycling model produced varied promoter-state fractions representing chromatin environments of quiescent-but-inducible HIV promoters in a range of biological contexts (Fig 1), and captured the observed differences in noise upon upstream signal activation (Fig 2).

Our previous experimental studies demonstrated that NF-κB target genes with distinct chromatin environments respond differently to TNF stimulation, resulting in different bursting dynamics and ultimately different patterns of inducible gene expression noise [4,28]. For example, we showed that by increasing histone acetylation via treatment with the HDAC inhibitor trichostatin A (TSA) at an HIV LTR promoter with a refractory chromatin environment, we could switch TNF from increasing burst frequency to increasing burst size, leading to increased gene activation noise [4]. Our model reproduced this result (Fig 7), while also allowing for exploration of how downstream steps in the transcriptional cycling model, such as pause release, also affected noise [72]. Interestingly, this result contrasts with a recent experimentally elegant study demonstrating that increasing histone acetylation via targeted recruitment of the histone acetyltransferase p300 decreased noise in gene expression following TF stimulation [73]. It is likely that by exploring different initial promoter states, our model would also be able to reproduce this result.

When we applied the three-state model coupled to positive feedback to simulate activation of latent-but-inducible HIV viral integrations, we found that it better represented our experimental observations of chromatin remodeling and RNAPII pause release [4]. Unlike the two-state random telegraph model, the transcriptional cycling model reproduced experimentally observed bimodal patterns of HIV expression within our parameter range. Furthermore, the range of initial promoter states captured by the transcriptional cycling model led to different patterns of gene expression noise following activation (i.e., a sudden change in a model parameter that increases the rate of transcription). This model result was consistent with our experimental observation that the same activating TF can influence multiple mechanisms of activation for our viral integrations, resulting in varied activation profiles. The results of this study highlight the importance of including chromatin remodeling within transcriptional bursting models, particularly when comparing target genes with large variations in initial promoter state fractions, representing different chromatin environments.

Several studies have demonstrated that Tat positive feedback is a direct contributor to bimodal HIV gene expression [69,71]. However, we observed that the parameter regions in our model that produced bimodal gene expression did not require Tat positive feedback. For our model assumptions, Tat positive feedback on PPRR further amplifies the separation between activated cells and non-activated cells, but the initial promoter state regulates bimodality. In settings with more homogeneous chromatin environments, positive feedback may contribute to the regulation of bimodality, but we did not explore these parameter regions in this study.

Gene expression noise has both intrinsic and extrinsic contributions to noise, with extrinsic noise referring to cell-to-cell differences that affect global gene expression in the cell, such as total levels of RNAPII, whereas intrinsic noise captures expression variability that varies stochastically by gene [23,74–76]. Our model analysis focused on intrinsic noise because we used stochastic simulations of gene expression while imposing uniform changes in upstream signaling inputs (e.g., increasing PPRR 2-fold). However, many studies have found a role for NF-κB signaling variations in regulation heterogeneity in gene expression [77–80]. Indeed, we have previously reported that transcriptional output is strongly correlated to the fold-change in upstream NF-κB signal activation in individual cells, regardless of initial chromatin state [81]. In future modeling studies, uniform fold-changes in rate parameters could be replaced by variable fold changes to represent extrinsic signaling variability, and the contribution of intrinsic versus extrinsic noise could be inferred by comparing the noise generated by stochastic simulation of the transcriptional cycling model to these results. If NF-κB is primarily assumed to increase polymerase binding and pause release rates, then we would expect extrinsic noise in NF-κB signaling to increase the variance between activated cells, similar to experimental reports. If NF-κB affects the burst initiation or termination rates, then the fractional responses of cells (i.e., bimodality) might also be affected, although this would depend on the initial chromatin states of the gene targets.

In our study, we used error minimization to choose initial parameter sets for our experimentally measured viral integrations, however these parameter sets are not unique. A limitation of our study is that there are many different parameter sets that would produce qualitatively similar results. Additional work would be required to fit the transcriptional cycling model to experimental data to identify unique parameter sets describing each viral integration. In particular, fitting the non-Poisson moments for RNA and protein distributions might provide additional information to further restrict the possible solutions for initializing the three-state transcriptional cycling model [7,82]. This could then be used in conjunction with techniques for fitting datasets, such as Bayesian parameter estimation, which requires the knowledge of appropriate priors from the solutions.

Future work with this model allows for studies of how exogeneous perturbations affecting chromatin remodeling combined with other transcriptional regulatory mechanisms (e.g. RNAPII pause release or transcription factor recruitment) synergize to activate gene expression. For example, using TSA to induce chromatin remodeling at the LTR promoter is a clinical strategy to reactivate latent HIV reservoirs to clear the infection [4], and this has been found to synergize with TNF and other NF-κB activators [4,83,84]. Using this model, we can more accurately simulate the effects of multiple drug treatments affecting these pathways and predict how they will impact gene expression noise and viral activation and provide possible avenues for future developments.

## Materials and methods

### Model development

The three-state transcriptional cycling model without positive feedback can be represented by the following mass-action kinetic equations:

$$\frac{d[UP]}{dt}=-BIR[UP]+BTR(\left[AP]+[BP\right])$$ Eq 4

$$\frac{d[AP]}{dt}=BIR[UP]-(BTR+PBR)[AP]+PPRR[BP]$$ Eq 5

$$\frac{d[BP]}{dt}=PBR[AP]-(BTR+PPRR)[BP]$$ Eq 6

$$\frac{d[RNA]}{dt}=PPRR[BP]-d_{rna}[RNA]$$ Eq 7

$$\frac{d[Protein]}{dt}=k_{p}[RNA]-d_{protein}[Protein]$$ Eq 8

While transcription is stochastic at the single-cell level, we assumed that gene expression follows a deterministic trajectory under population dynamics. We also assumed that the promoter can exist in one of three states, UP, AP, or BP. This conservation of states can be expressed by the following equation.


[UP]+[AP]+[BP]=1 Eq 9

These equations were deterministically solved using an ODE solver. We utilized the ODE solver in NFSIM [85] to model our system at steady-states conditions a time interval of ten days, capturing dynamics at equal time increments. We used a parameter set that could capture the wide range of mRNA responses, PBR = [0.1 0.5 1 5 10 50 100 500] hr^-1^, PPRR = [0.1 0.5 1 5 10 50 100] hr^-1^, BIR = [0.005 0.01 0.05 0.1 0.5 1 5 10 50] hr^-1^, and BTR = [0.005 0.01 0.05 0.1 0.5 1 5 10 50] hr^-1^. These ranges are up to 10-fold higher and/or lower than the parameter ranges used for our analysis of inducible transcription (see Table 1), because we wished to explore bursting dynamics across a wide range of constitutive transcriptional activity prior to focusing on inducible transcription. Data matrices of the solutions were analyzed using MATLAB.

### Stochastic simulation and activation

To stochastically simulate the model, we used the SSA utility of NFSIM [85], which implements Gillespie algorithm [86]. The model was first initialized under steady-state basal conditions for ten days. The system was then activated by increasing PPRR two-fold at time = 0, and then simulated out for 24 hours. The promoter states, along with RNA and protein counts, were captured in equal time increments. Unless otherwise noted, each parameter combination was stochastically simulated 1,000 times. To classify these simulations, we tested deviance from unimodality of the probability density distribution utilizing the Hartigan’s dip test [87,88], with dip > 0.05, and calculated the p value null hypothesis of unimodal distribution < 0.15. Simulations that met these criteria were classified as “bimodal.” For the remainder of the simulations, we separated “always on” from “always off” with a threshold value of 250 proteins, in line with that used in our previous study to classify T cells expressing HIV (“on” or activated) and not expressing HIV (“off” or not activated) [49].

At each relevant time point, cell-population averages and Fano factors for mRNA counts and protein counts were calculated from the single-cell simulations, with 95% confidence intervals for these metrics determined via bootstrapping (n = 10,000 and α = 0.05) as described previously [28]. Trajectories, heatmaps, bar plots, pie charts, violin plots, and kernel estimations were then generated in MATLAB to meaningfully visualize these datasets. Other simulations involving protein feedback and multi-activation steps used the same parameter combinations and analysis.

To visualize the differences along the promoter accessibility and the transcriptional cycling ratios, six different parameter combinations were selected. Three represented variations in promoter accessibility (BIR:BTR = 0.1, 1, or 10), while the other three represented variations in transcriptional cycling (PPRR:PBR = 0.1, 1, or 10) (Table 4).

**Table 4:** Ratio Parameter Descriptions.

Name	BIR	BTR	PBR	PPRR
BIR:BTR = 0.1	0.01 hr^-1^	0.1 hr^-1^	10 hr^-1^	10 hr^-1^
BIR:BTR = 1	0.1 hr^-1^	0.1 hr^-1^	10 hr^-1^	10 hr^-1^
BIR:BTR = 10	1 hr^-1^	0.1 hr^-1^	10 hr^-1^	10 hr^-1^
PPRR:PBR = 0.1	0.1 hr^-1^	0.1 hr^-1^	10 hr^-1^	1 hr^-1^
PPRR:PBR = 1	0.1 hr^-1^	0.1 hr^-1^	10 hr^-1^	10 hr^-1^
PPRR:PBR = 10	0.1 hr^-1^	0.1 hr^-1^	10 hr^-1^	100 hr^-1^

### Fitting parameters to experimental data

We fit our model to a previously published experimental data set measured in four Jurkat T cell lines each containing a latent HIV virus integrated at a unique location in the genome [4]. This data set consisted of measurements of HIV transcript distributions in these four cell populations in the basal state and following 2 and 4 hours of stimulation with TNF in the presence of cycloheximide to protein production and thus Tat positive feedback [4]. We used several assumptions and biological considerations to limit the range of the parameter space that we searched.

We first limited the parameter space to parameters that meet conditions for bursting transcription, characterized by infrequent transitions from low levels of mRNA production to high levels of mRNA production. Based on our steady-state analysis, this would result in higher levels of BP as compared to AP and BP, and is met with the following condition:

BTR≥BIR Eq 10

The second condition is imposed to reproduce the experimentally observed differences in our T cell populations with latent HIV integrations that are low activating (integrations 8.4 and 4.4) and high activation (integrations 6.6 and 10.6). The high-activating viral integrations have higher levels of histone acetylation at the promoter under basal conditions, while the low-activating viral integrations have higher levels of histone occupancy. To correlate this to our three-state transcriptional cycling model, the low-activating viral integrations should have higher levels of UP as compared to the high-activating viral integrations. We know that the amount of UP is controlled by the BIR:BTR ratio, so we can represent the parameter space as follows:

$$\frac{BTR_{LA}}{BIR_{LA}}>\frac{BTR_{HA}}{BIR_{HA}}$$ Eq 11

Our third condition is imposed to reproduce the observation that high-activating viral integrations have higher levels of open chromatin, without significant changes to the amount of transcriptional machinery priming as compared to the low-activation viral integrations. We can capture this in our model if AP is greater for high-activating vs. low-activating viral integrations, whereas the amount of BP remains the same. Based on our steady-state analysis of PBR to PPRR, we can capture with the following condition:

$$\frac{PPRR_{LA}}{PBR_{LA}}<\frac{PPRR_{HA}}{PBR_{HA}}$$ Eq 12

With these conditions, we then sought to find the parameter space that best matched experimental results. Stochastic simulations were run over a biologically plausible parameter space (defined as in Table 1), where counts of basal mRNA < 3 as determined through deterministic solutions and bimodality was observed according to our bimodal criteria described above. We then used the three considerations above to select appropriate parameter spaces for each viral integration. For each of these chosen parameter combinations, 1,000 stochastic simulations were run, with promoter states, mRNA, and protein data recorded out to 24 hours. To quantify the match, we performed root mean square error (MSE) minimization over mRNA transcript counts, as well as Fano factor and CV for the experimental data at basal, 2, and 4 hours for the four viral integrations. The best fits were defined by the parameter set with the lowest combined MSE across each feature. Finally, activation trajectories of mRNA and protein were then recorded out to 24 hours using the activation schemes as described and in the presence of Tat positive feedback for 1,000 simulations.

## Supporting information

S1 Fig Steady-state heatmaps and PPRR:BTR comparisons (related to Fig 1). (A-C) Heat maps of the deterministic steady state solutions for UP, AP, and BP fractional probabilities. Parameter ranges are represented low to high via arrow directionality and correspond to the following sets: PBR = [0.1 0.5 1 5 10 50 100 500] hr^-1^, PPRR = [0.1 0.5 1 5 10 50 100] hr^-1^, BIR = [0.005 0.01 0.05 0.1 0.5 1 5 10 50] hr^-1^, and BTR = [0.005 0.01 0.05 0.1 0.5 1 5 10 50] hr^-1^. (D) Deterministic solution of mRNA counts representing steady state values plotted as a log scale heatmap. Parameter ranges are represented low to high via arrow directionality and correspond to the following sets: PBR = [0.1 0.5 1 5 10 50 100 500] hr^-1^, PPRR = [0.1 0.5 1 5 10 50 100] hr^-1^, BIR = [0.005 0.01 0.05 0.1 0.5 1 5 10 50] hr^-1^, and BTR = [0.005 0.01 0.05 0.1 0.5 1 5 10 50] hr^-1^. Square inset corresponds to the following parameter set: PBR = 10 hr^-1^, BIR = 0.1 hr^-1^, PPRR = [0.5 1 5 10 50] hr^-1^, and BTR = [0.01 0.05 0.1 0.5 1] hr^-1^. (E-F) Pie charts of the fractional promoter-state probability (E) and Fano factor (F) at each parameter combination. Values determined by stochastic simulation under basal conditions out to 10 days for 1,000 cells for each parameter combination. Promoter states denoted as UP (blue), AP (teal), and BP (yellow).
(PDF)

S2 Fig Classification of phenotypes after activation via PPRR increase (related to Fig 2). (A) Fold Change mRNA trajectories for PPRR increases of 0.5 (green), 2 (cerulean), 5 (red), 10 (blue), and 50 (purple). Shaded areas are 95% confidence intervals. PPRR and PBR were set at 10 hr^-1^. (B) Average Protein counts at 24 hours. Protein counts were generated through stochastic simulation for 1,000 cells for each parameter combination. Phenotypes of “Always On,” “Bimodal,” and “Always Off” were established by separating out the bimodal population (defined by Hartigan’s Dip Value > 0.05 and a p-test < 0.15 (See Materials and Methods for more information). (C) Bar chart representing the percentage of each promoter state from basal initialization conditions, separated by ending phenotype classification. Error bars represent 95% bootstrapped confidence intervals.
(PDF)

S3 Fig Feedback strength influence on fractional state probabilities and protein counts (related to Fig 3). (A-B) Heatmap of average mRNA at 24 hours (A) and fractional promoter-state probabilities in the initial state (B) for a range of feedback strengths. All other parameters are set to BIR = 0.1 hr^-1^, BTR = 1 hr^-1^, PBR = PPRR = 10 hr^-1^. Data was generated by stochastic simulation for 1,000 cells for each parameter combination. The feedback terms K (half max) and A (amplification factor) were varied over 5 orders of magnitude. (C) Feedback strength calculated for varied K values and plotted versus protein.
(PDF)

S4 Fig Model fits of experimental HIV data compared to simulations using computational PPRR activation scheme (related to Fig 5). (A-C) Scatterplots of results from simulation of the three-state transcriptional cycling model without feedback compared to experimental measurements of mRNA distributions from populations of T cells harboring latent HIV integrations and stimulated with TNF with Tat feedback blocked (from ref. 4). Correlation between simulation and experimental data is shown for mRNA average (A), Fano factor (B), and CV (C). Error bars represent 95% bootstrapped confidence intervals.
(PDF)

S5 Fig Model fits of experimental HIV data compare to simulations using computational multi-step activation scheme (related to Fig 6). (A-C) Scatterplots of results from simulation of the three-state transcriptional cycling model without feedback compared to experimental measurements of mRNA distributions from populations of T cells harboring latent HIV integrations and stimulated with TNF with Tat feedback blocked (from ref. 4). Correlation between simulation and experimental data is shown for mRNA average (A), Fano factor (B), and CV (C). Error bars represent 95% bootstrapped confidence intervals.
(PDF)

S6 Fig Comparisons of average mRNA and Fano factor across activation paths (related to Fig 7). (A-B) Average mRNA counts (A) and Fano factor (B) for the four activation paths across the four HIV integrations for timepoints of 0, 1, 2, 4 and 24 hours. mRNA counts were generated through stochastic simulation for 1,000 cells for each parameter combination. Error bars represent 95% bootstrapped confidence intervals. (C) Percentage of cells above ‘ON’ threshold (set at 250 proteins per cell) at 24 hours.
(PDF)
