# Supplementary material for: A transcriptional cycling model recapitulates chromatin-dependent features of noisy inducible transcription
Source: PLoS Comput Biol. 2022 Sep 9;18(9):e1010152. doi: 10.1371/journal.pcbi.1010152 (PMC9491597; doi:10.1371/journal.pcbi.1010152)
Supplement: S1 Fig — (A-C) Heat maps of the deterministic steady state solutions for UP, AP, and BP fractional probabilities. Parameter ranges are represented low to high via arrow directionality and correspond to the following sets: PBR = [0.1 0.5 1 5 10 50 100 500] hr-1, PPRR = [0.1 0.5 1 5 10 50 100] hr-1, BIR = [0.005 0.01 0.05 0.1 0.5 1 5 10 50] hr-1, and BTR = [0.005 0.01 0.05 0.1 0.5 1 5 10 50] hr-1. (D) Deterministic solution of mRNA counts representing steady state values plotted as a log scale heatmap. Parameter ranges are represented low to high via arrow directionality and correspond to the following sets: PBR = [0.1 0.5 1 5 10 50 100 500] hr-1, PPRR = [0.1 0.5 1 5 10 50 100] hr-1, BIR = [0.005 0.01 0.05 0.1 0.5 1 5 10 50] hr-1, and BTR = [0.005 0.01 0.05 0.1 0.5 1 5 10 50] hr-1. Square inset corresponds to the following parameter set: PBR = 10 hr-1, BIR = 0.1 hr-1, PPRR = [0.5 1 5 10 50] hr-1, and BTR = [0.01 0.05 0.1 0.5 1] hr-1. (E-F) Pie charts of the fractional promoter-state probability (E) and Fano factor (F) at each parameter combination. Values determined by stochastic simulation under basal conditions out to 10 days for 1,000 cells for each parameter combination. Promoter states denoted as UP (blue), AP (teal), and BP (yellow). (PDF) [file pcbi.1010152.s001.pdf]

S1 Figure

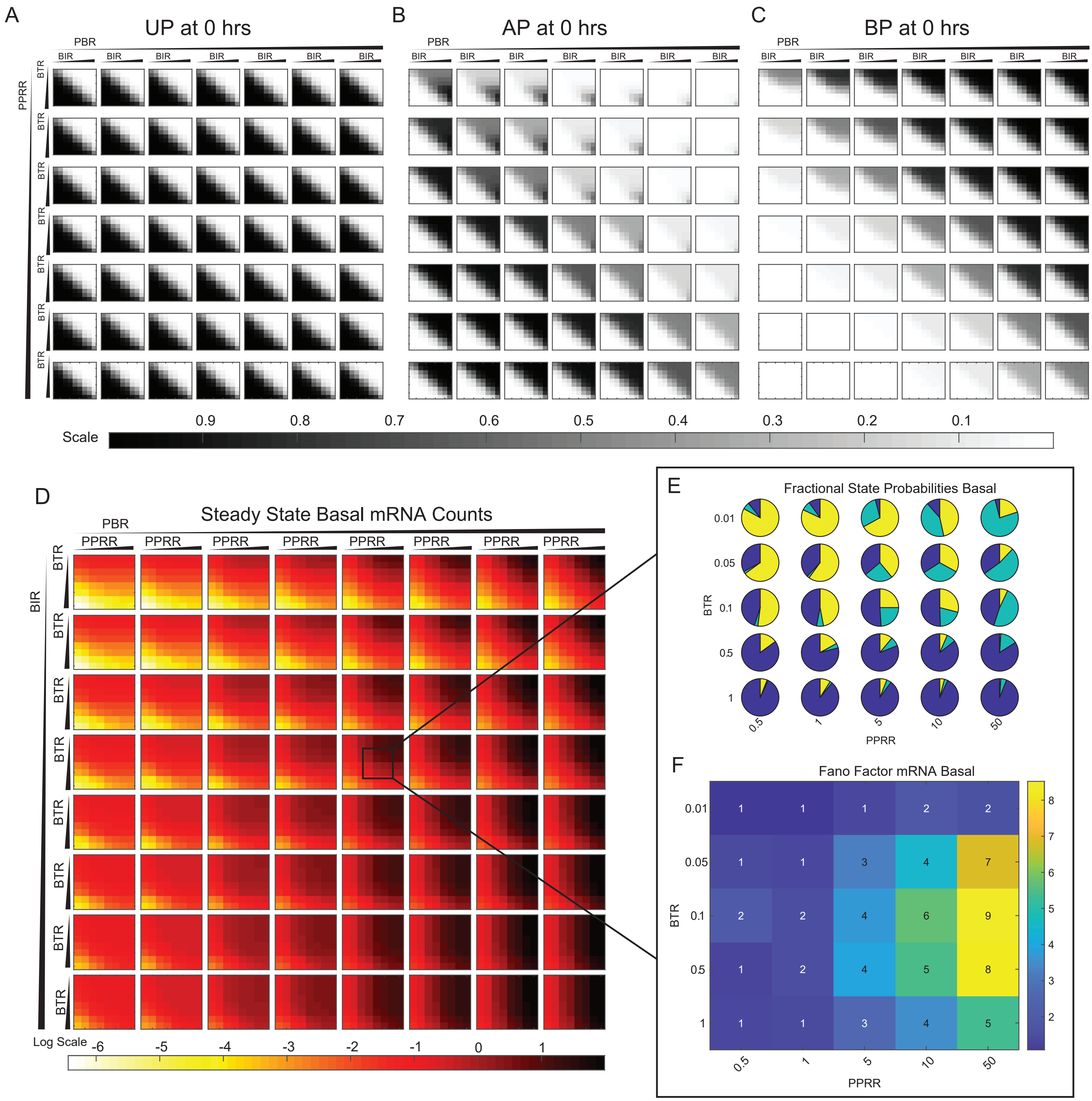

S1 Fig. Steady-state heatmaps and PPRR:BTR comparisons (related to Fig 1).
(A-C) Heat maps of the deterministic steady state solutions for UP, AP, and BP fractional probabilities. Parameter ranges are represented low to high via arrow directionality and correspond to the following sets: PBR=[0.1 0.5 1 5 10 50 100 500] hr-1, PPRR=[0.1 0.5 1 5 10 50 100] hr-1, BIR=[0.005 0.01 0.05 0.1 0.5 1 5 10 50] hr-1, and BTR=[0.005 0.01 0.05 0.1 0.5 1 5 10 50] hr-1. (D) Deterministic solution of mRNA counts representing steady state values plotted as a log scale heatmap. Parameter ranges are represented low to high via arrow directionality and correspond to the following sets: PBR=[0.1 0.5 1 5 10 50 100 500] hr-1, PPRR=[0.1 0.5 1 5 10 50 100] hr-1, BIR=[0.005 0.01 0.05 0.1 0.5 1 5 10 50] hr-1, and BTR=[0.005 0.01 0.05 0.1 0.5 1 5 10 50] hr-1. Square inset corresponds to the following parameter set: PBR=10 hr-1, BIR=0.1 hr-1, PPRR=[0.5 1 5 10 50] hr-1, and BTR=[0.01 0.05 0.1 0.5 1] hr-1. (E-F) Pie charts of the fractional promoter-state probability (E) and Fano factor (F) at each parameter combination. Values determined by stochastic simulation under basal conditions out to 10 days for 1,000 cells for each parameter combination. Promoter states denoted as UP (blue), AP (teal), and BP (yellow).
