# Supplementary material for: A transcriptional cycling model recapitulates chromatin-dependent features of noisy inducible transcription
Source: PLoS Comput Biol. 2022 Sep 9;18(9):e1010152. doi: 10.1371/journal.pcbi.1010152 (PMC9491597; doi:10.1371/journal.pcbi.1010152)
Supplement: S2 Fig — (A) Fold Change mRNA trajectories for PPRR increases of 0.5 (green), 2 (cerulean), 5 (red), 10 (blue), and 50 (purple). Shaded areas are 95% confidence intervals. PPRR and PBR were set at 10 hr-1. (B) Average Protein counts at 24 hours. Protein counts were generated through stochastic simulation for 1,000 cells for each parameter combination. Phenotypes of “Always On,” “Bimodal,” and “Always Off” were established by separating out the bimodal population (defined by Hartigan’s Dip Value > 0.05 and a p-test < 0.15 (See Materials and Methods for more information). (C) Bar chart representing the percentage of each promoter state from basal initialization conditions, separated by ending phenotype classification. Error bars represent 95% bootstrapped confidence intervals. (PDF) [file pcbi.1010152.s002.pdf]

S2 Figure

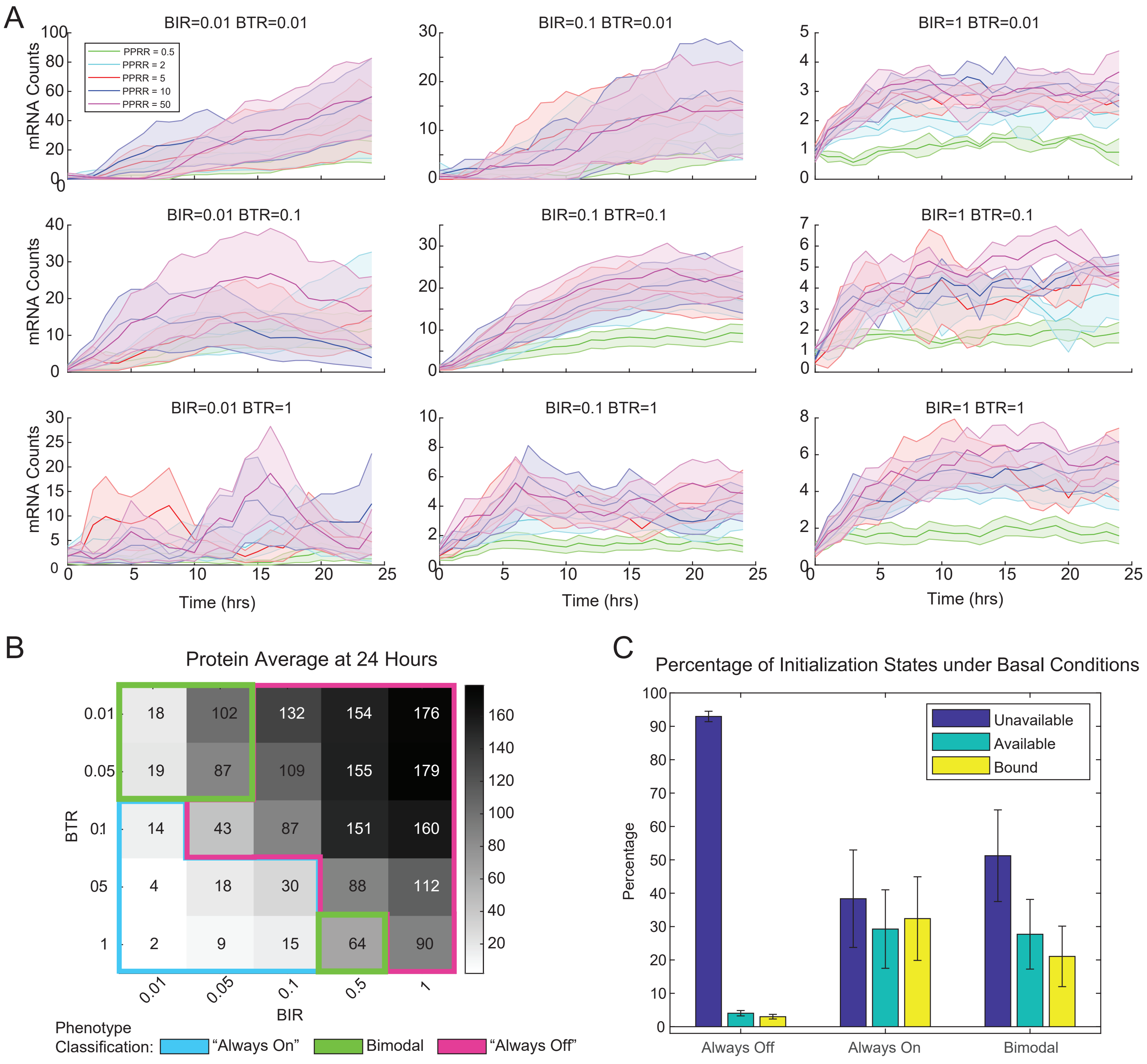

S2 Fig Classification of phenotypes after activation via PPRR increase (related to Fig 2). (A) Fold Change mRNA trajectories for PPRR increases of 0.5 (green), 2 (cerulean), 5 (red), 10 (blue), and 50 (purple). Shaded areas are 95% confidence intervals. PPRR and PBR were set at 10 hr^{-1}. (B) Average Protein counts at 24 hours. Protein counts were generated through stochastic simulation for 1,000 cells for each parameter combination. Phenotypes of "Always On," "Bimodal," and "Always Off" were established by separating out the bimodal population (defined by Hartigan's Dip Value > 0.15 and a p-test < 0.05 (see Materials and Methods for more information). (C) Bar chart representing the percentage of each promoter state from basal initialization conditions, separated by ending phenotype classification. Error bars represent 95% bootstrapped confidence intervals.
