# Supplementary material for: A transcriptional cycling model recapitulates chromatin-dependent features of noisy inducible transcription
Source: PLoS Comput Biol. 2022 Sep 9;18(9):e1010152. doi: 10.1371/journal.pcbi.1010152 (PMC9491597; doi:10.1371/journal.pcbi.1010152)
Supplement: S3 Fig — (A-B) Heatmap of average mRNA at 24 hours (A) and fractional promoter-state probabilities in the initial state (B) for a range of feedback strengths. All other parameters are set to BIR = 0.1 hr-1, BTR = 1 hr-1, PBR = PPRR = 10 hr-1. Data was generated by stochastic simulation for 1,000 cells for each parameter combination. The feedback terms K (half max) and A (amplification factor) were varied over 5 orders of magnitude. (C) Feedback strength calculated for varied K values and plotted versus protein. (PDF) [file pcbi.1010152.s003.pdf]

S3 Figure

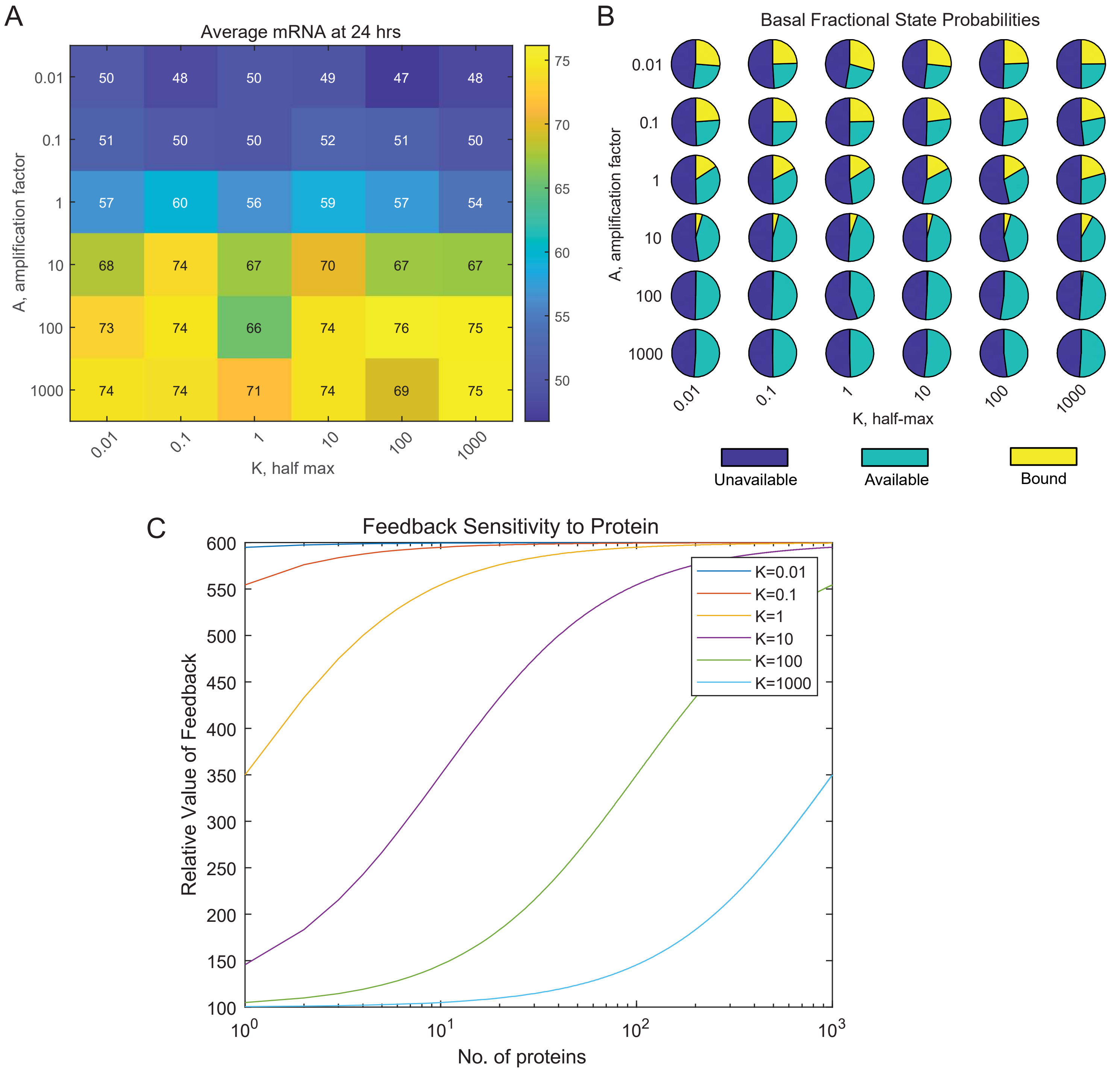

S3 Fig Feedback strength influence on fractional state probabilities and protein counts (related to Fig 3)

(A-B) Heatmap of average mRNA at 24 hours (A) and fractional promoter-state probabilities in the initial state (B) for a range of feedback strengths. All other parameters are set to : BIR = 0.1 hr^{-1}, BTR = 1 hr^{-1}, PBR = PPRR = 10 hr^{-1}. Data was generated by stochastic simulation for 1,000 cells for each parameter combination. The feedback terms K (half max) and A (amplification factor) were varied over 5 orders of magnitude. (C) Feedback strength calculated for varied K values and plotted versus protein.
