# Supplementary material for: A transcriptional cycling model recapitulates chromatin-dependent features of noisy inducible transcription
Source: PLoS Comput Biol. 2022 Sep 9;18(9):e1010152. doi: 10.1371/journal.pcbi.1010152 (PMC9491597; doi:10.1371/journal.pcbi.1010152)
Supplement: S5 Fig — (A-C) Scatterplots of results from simulation of the three-state transcriptional cycling model without feedback compared to experimental measurements of mRNA distributions from populations of T cells harboring latent HIV integrations and stimulated with TNF with Tat feedback blocked (from ref. 4). Correlation between simulation and experimental data is shown for mRNA average (A), Fano factor (B), and CV (C). Error bars represent 95% bootstrapped confidence intervals. (PDF) [file pcbi.1010152.s005.pdf]

S5 Figure

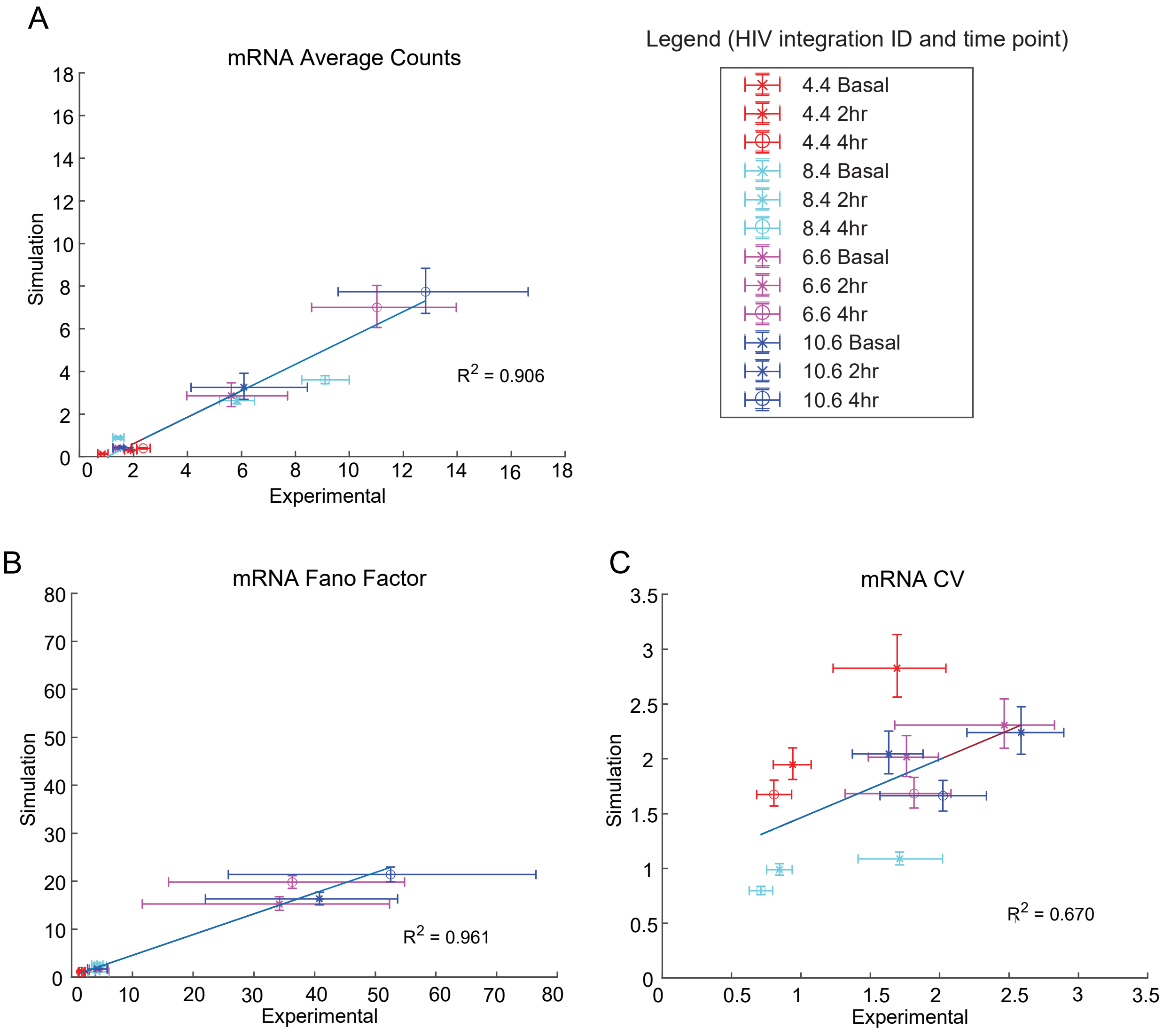

S5 Fig. Model fits of experimental HIV data compare to simulations using computational multi-step activation scheme (related to Fig 6).

(A-C) Scatterplots of results from simulation of the three-state transcriptional cycling model without feedback compared to experimental measurements of mRNA distributions from populations of T cells harboring latent HIV integrations and stimulated with TNF with Tat feedback blocked (from ref. 4). Correlation between simulation and experimental data is shown for mRNA average counts (A), Fano factor (B), and CV (C). Error bars represent 95% bootstrapped confidence intervals.
