# Supplementary material for: A transcriptional cycling model recapitulates chromatin-dependent features of noisy inducible transcription
Source: PLoS Comput Biol. 2022 Sep 9;18(9):e1010152. doi: 10.1371/journal.pcbi.1010152 (PMC9491597; doi:10.1371/journal.pcbi.1010152)
Supplement: S6 Fig — (A-B) Average mRNA counts (A) and Fano factor (B) for the four activation paths across the four HIV integrations for timepoints of 0, 1, 2, 4 and 24 hours. mRNA counts were generated through stochastic simulation for 1,000 cells for each parameter combination. Error bars represent 95% bootstrapped confidence intervals. (C) Percentage of cells above ‘ON’ threshold (set at 250 proteins per cell) at 24 hours. (PDF) [file pcbi.1010152.s006.pdf]

S6 Figure

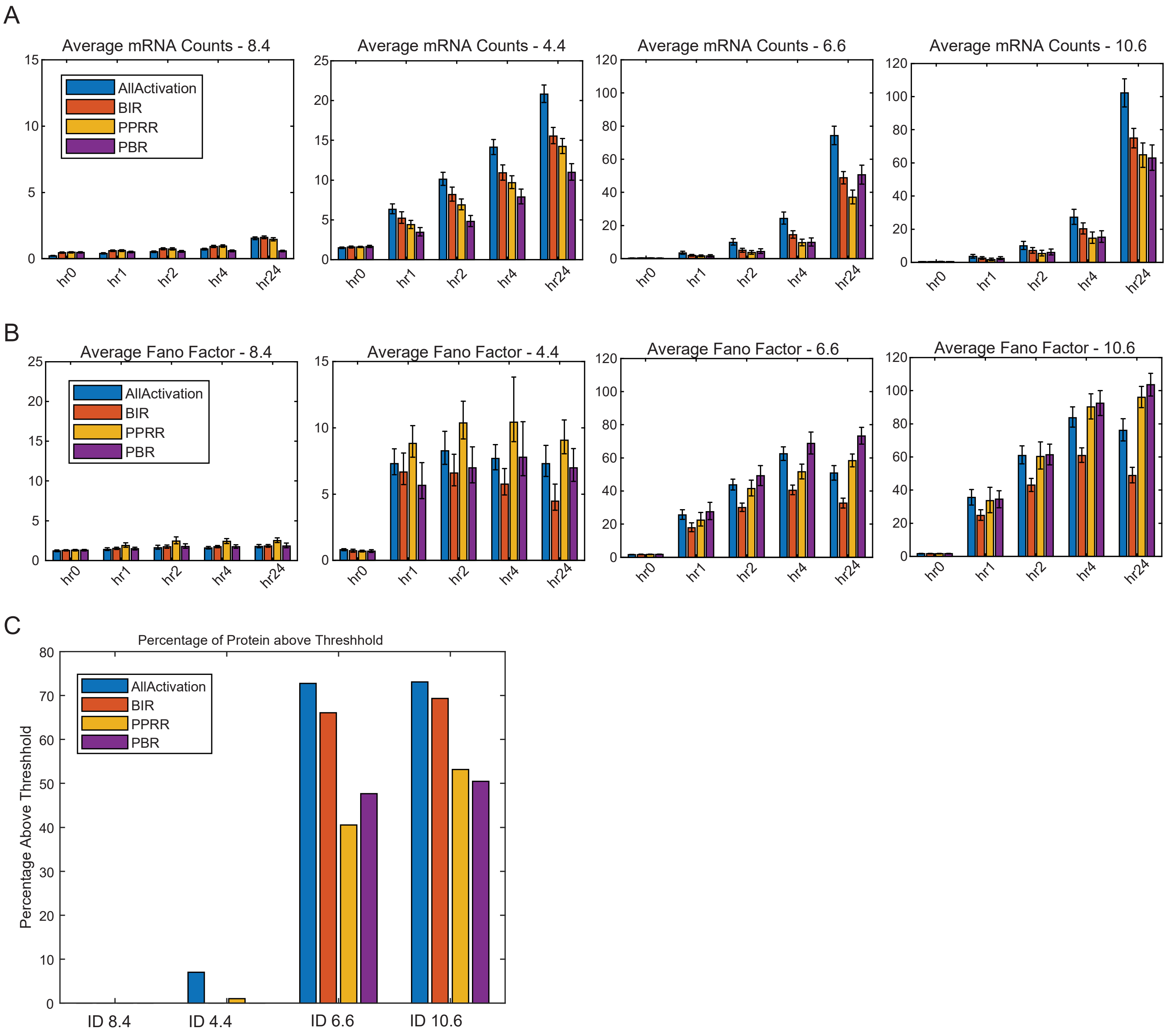

S6 Fig. Comparisons of average mRNA and Fano factor across activation paths (related to Fig 7). (A-B) Average mRNA counts (A) and Fano factor (B) for the four activation paths across the four HIV integrations for timepoints of 0, 1, 2, 4 and 24 hours. mRNA counts were generated through stochastic simulation for 1,000 cells for each parameter combination. Error bars represent 95% bootstrapped confidence intervals. (C) Percentage of cells above 'ON' threshold (set at 250 proteins per cell) at 24 hours.
